# Horizontally-acquired genetic elements in the mitochondrial genome of a centrohelid *Marophrys* sp. SRT127

**DOI:** 10.1038/s41598-019-41238-6

**Published:** 2019-03-19

**Authors:** Yuki Nishimura, Takashi Shiratori, Ken-ichiro Ishida, Tetsuo Hashimoto, Moriya Ohkuma, Yuji Inagaki

**Affiliations:** 1 Graduate School of Life and Environmental Sciences, University of Tsukuba, Tsukuba, Japan; 2 Center for Computational Sciences, University of Tsukuba, Tsukuba, Japan; 3 RIKEN BioResource Research Center, Japan Collection of Microorganisms Microbe Division, Tsukuba, Japan; 4 Present Address: RIKEN BioResource Research Center, Japan Collection of Microorganisms Microbe Division, Tsukuba, Japan; 5 Present Address: Japan Agency for Marine-Earth Science and Technology (JAMSTEC), Yokosuka, Japan

## Abstract

Mitochondrial genomes exhibit diverse features among eukaryotes in the aspect of gene content, genome structure, and the mobile genetic elements such as introns and plasmids. Although the number of published mitochondrial genomes is increasing at tremendous speed, those of several lineages remain unexplored. Here, we sequenced the complete mitochondrial genome of a unicellular heterotrophic eukaryote, *Marophrys* sp. SRT127 belonging to the Centroheliozoa, as the first report on this lineage. The circular-mapped mitochondrial genome, which is 113,062 bp in length, encodes 69 genes typically found in mitochondrial genomes. In addition, the *Marophrys* mitochondrial genome contains 19 group I introns. Of these, 11 introns have genes for homing endonuclease (HE) and phylogenetic analyses of HEs have shown that at least five *Marophrys* HEs are related to those in green algal plastid genomes, suggesting intron transfer between the *Marophrys* mitochondrion and green algal plastids. We also discovered a putative mitochondrial plasmid in linear form. Two genes encoded in the circular-mapped mitochondrial genome were found to share significant similarities to those in the linear plasmid, suggesting that the plasmid was integrated into the mitochondrial genome. These findings expand our knowledge on the diversity and evolution of the mobile genetic elements in mitochondrial genomes.

## Introduction

Mitochondria have emerged by the endosymbiosis between the last common ancestor of eukaryotes and an α-proteobacterium. All extent eukaryotes have mitochondria or mitochondrial remnant organelles, with only one exception of *Monocercomonoides* sp. PA203^1^ (now classified as *Monocercomonoides exilis*^2^). Although most of the genes in the α-proteobacterium that gave rise to the ancestral mitochondrion have been lost or transferred to the host nucleus during organellogenesis, typical mitochondria, which can carry out aerobic respiration, still retain their genomes^3^ (mitochondrial genomes, mtDNAs). The gene repertories in mtDNAs have differently reduced in individual lineages in eukaryotes from the ancestral mtDNA that encoded at least about 100 genes for oxidative phosphorylation, translation, transcription, protein transport, protein maturation and RNA processing as found in mtDNA of jakobids^4,5^. In contrast, apicomplexan parasites and their relatives have only three to five genes in their mtDNAs^6^. mtDNAs also vary with regard to genome architecture, from a simple monocircular molecule (e.g., human mtDNA) to a complex network comprising thousands of chromosomes (e.g., mtDNAs in kinetoplastids). Monolinear mtDNAs were reported from separate branches of eukaryotes as well as mtDNAs consisting of multiple linear chromosomes^7–11^. Likewise, the size range of mtDNA is broad among eukaryotes. Apicomplexan parasites are known to have the smallest mtDNAs, approximately 6 Kb in size^8^. On the other hand, land plants tend to contain large-sized mtDNAs, up to 11 Mb in *Silene conica*^12^.

mtDNAs of extant species vary markedly in terms of their gene repertories, genome structure, and genome size, as described above. On top of that, mobile genetic elements confer additional layers of mtDNA diversity. Group I introns and group II introns are the examples of the mobile genetic elements found in bacterial, mtDNA and plastid genomes^13^. Group I and group II introns are also known to be able to catalyze self-splicing by forming distinct structures, which enable us to distinguish two types of introns from DNA sequences: Group I introns form the ribozyme structure consisting of 10 stem-loops^14^, while group II introns require a characteristic secondary structure, such as a central wheel with six stems, for the self-splicing reaction^15^. Both of group I and group II introns show patchy distributions, which are considered to be formed by intron invasion from an intron-containing locus to a homologous, but intron-less locus in the same species and/or a distantly related organisms^16,17^. Group I intron transfers are facilitated by homing endonucleases (HEs) encoded by introns themselves (Note that HEs can act as maturases that facilitate intron splicing as well^13^). The HEs introduce a double-strand break in the recipient (intron-less) allele that leads to the homologous recombination between intron-containing and intron-less alleles. Individual HEs possess distinct specificities for the nucleotide sequences to recognize and digest. Therefore, a particular HE can introduce the corresponding intron only into a certain position in a genome^16,18^. Conversely, the group I introns (and the corresponding HEs) found in the homologous position in different genomes are expected to share their evolutionary origin^19–21^. The mechanism of group II intron transfer is different from that of group I introns, but intron-encoded proteins and their sequence recognition specificities play a central role in invasion for group II intron, as in group I intron. Thus, the evolutionary history of group II introns can be retraced by analyzing intron positions and intron-encoded proteins^22,23^.

Another type of mobile genetic elements in mitochondria is the linear plasmids^24^. Typical linear plasmids in mitochondria contain terminal inverted repeats and one or some open reading frames (ORFs) usually encoding virus-type DNA and/or RNA polymerases, designated as *dpo* and *rpo*, respectively, suggesting their independent replication and transcription^25^. Linear plasmids are extrachromosomal elements in mitochondria, but can occasionally integrate into mtDNA through recombination^26^. To date, linear plasmids in mitochondria or plasmid-derived *dpo*/*rpo* sequences in mtDNA have been reported from fungi^27^, a slime mold^28^, plants^24^ and a ciliate^29^. Consistent with the genetic mobility proposed for the linear plasmids, the phylogenies of *dpo*/*rpo* were found to be inconsistent with the organismal phylogeny^30^.

Heliozoa was traditionally defined as a taxonomic group of the axopodium-bearing, heterotrophic unicellular eukaryotes mainly living in freshwater environments^31^. At present, Heliozoa comprises two classes, namely Endohelea and Centrohelea^32,33^. The phylogenetic position of Heliozoa in the eukaryotes has been controversial for a long time, but recent phylogenomic study suggests that Heliozoa and haptophytes form a monophyletic clade, Haptista, as a sister to the supergroup consisting of stramenopiles, alveolates, and Rhizaria^34–36^. Although data on the nuclear-encoded genes have been accumulated for heliozoans, as far as we know, no complete mtDNA sequence has been obtained from any member belonging to this group.

Here, we first present the complete mtDNA of a member of the Centrohelea, *Marophrys* sp. strain SRT127. The circular-mapped mtDNA of *Marophrys* contains 69 typical mtDNA-encoded genes. Up to 20 group I introns were found in six out of the 69 genes, and phylogenetic analyses of the corresponding HEs suggested that at least five introns share their origins with those in green algal plastid genomes. We also identified a linear plasmid carrying genes encoding *dpo* and *rpo*. The plasmid is likely to localize in mitochondrion because both mtDNA and plasmid share a deviant genetic code in which UGA codon assigns tryptophan. We further provide the evidence for the linear plasmid being integrated into the mtDNA. These findings expand our knowledge of the diversity of mobile genetic elements in mtDNAs.

## Results and Discussion

### Mitochondrial genome overview

**Figure 1:**
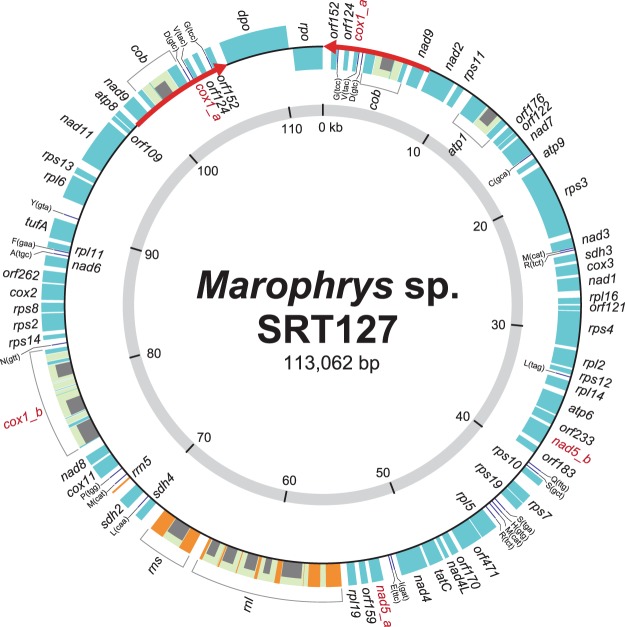
Mitochondrial genome of *Marophrys* sp. SRT127. Protein-coding genes and rRNA genes are shown in the cyan and orange boxes, respectively. Introns are indicated by light green and genes for homing endonuclease (HE) in introns are shown in the gray boxes. The genes for tRNA are depicted as black lines. Two red arrows indicate inverted repeat regions.

The mtDNA of a centrohelid *Marophrys* sp. SRT127 is 113,062 bp in length and mapped as circular (Fig. 1). The G + C contents of the mtDNA is 44.7%. It contains 42 kinds of protein-coding genes, which have been vertically inherited from the ancestor of all mitochondria. These 42 protein-coding genes include those for translation elongation factor (*tufA*) and the subunit of cytochrome *c* oxidase assembly (*cox11*), which are notable as most of the mtDNAs do not retain these genes except those in a limited number of eukaryotes^4,37–42^. In addition, the *Marophrys* mtDNA includes genetically mobile genes, namely those of *dpo*, *rpo* and 11 intronic HEs (see below). The *Marophrys* mtDNA also contains 12 functionally unidentified open reading frames (ORFs) longer than 100 amino acid residues. Large and small subunits of rRNA genes plus 5S rRNA gene were detected. We identified twenty kinds of genes for tRNAs that could translate 50 codons that cover 17 amino acids in total. During our BLAST analyses of the *Marophrys* mtDNA sequence against NCBI nr database, we observed UGA at the conserved tryptophan positions in the putative amino acid sequences. Thus, we concluded that UGA in the *Marophrys* mtDNA is used as tryptophan codon, instead of the termination signal for translation. Genes of the tRNAs that bind the codons UUA for leucine, UGA for tryptophan, ACN for threonine, AAR for lysine and CGN for arginine (R = A or G; N = A, C, G or T) were not found (Table S1). Genes coding for the RNA components for tmRNA and RNAase P could not be detected.

### Overview of the introns in the *Marophrys* mtDNA

In the *Marophrys* mtDNA, the genes for cytochrome oxidase *c* subunit 1 (*cox1*), apocytochrome *b* (*cob*), ATP synthetase subunit 1 (*atp1*), and large and small subunits of rRNA (*rnl* and *rns*) appeared to be split by a single or up to nine introns. The sequence motif shared among the canonical group I introns was detected from all of the introns in the *Marophrys* mtDNA (Table 1). A series of RT-PCR experiments confirmed that all of the introns were removed from the mature mRNAs (Fig. S1A). Henceforth here, we designate the introns found in the *Marophrys* mtDNA and those of the HEs harbored in the introns as follows. Introns are designated by adding ‘_i’ (intron) to their host gene names with the ascending numbers from the 5′ terminus (e.g., *rnl*_i1 and *rnl_*i2 are the first and second introns in the *rnl* gene, respectively). The HEs are distinct from each other by adding the corresponding superscripted host intron names (e.g., HEs harbored in *rnl_*i3 and *rnl_*i4 are designated as HE^*rnl_*i3^ and HE^*rnl_*i4^, respectively).

**Table 1:** Characteristics of introns in the *Marophrys* mtDNA.

Name	Length (bp)	Intron type^a^	HE type^b^	Shares the ancestry with introns in organellar genomes in green algae?^c^
*atp1*_i1	1,336	group IB	LAGLIDADG_1	Yes
*cob*_i1	606	group ID	ND	−^f^
*cob*_i2	1,351	group IB	LAGLIDADG_2^d^	No
*cox1*_i1	1,373	group IB	LAGLIDADG_1^d^	No
*cox1*_i2	381	group IB	ND	−^f^
*cox1*_i3	1,538	group ID	LAGLIDADG_1^d,e^	No
*cox1*_i4	258	group IB	ND	−^f^
*cox1*_i5	430	group IB	ND	−^f^
*cox1*_i6	1,156	group IB	LAGLIDADG_1	No
*cox1*_i7	462	group IB	ND	−^f^
*rnl*_i1	381	group IB	ND	−^f^
*rnl_i*2	297	group IB	ND	−^f^
*rnl*_i3	1,501	group IA3	LAGLIDADG_1	Yes
*rnl*_i4	1,137	group IB	LAGLIDADG_2	Yes
*rnl*_i5	1,177	group IA	LAGLIDADG_2^d,e^	No
*rnl*_i6	1,138	group IB	LAGLIDADG_2	Yes
*rnl*_i7	279	group IB	ND	−^f^
*rnl*_i8	796	group IB2	LAGLIDADG_1	Yes
*rns*_i1	1,260	group IA	LAGLIDADG_2	Yes

^a^Predicted by RNAweasel and Infernal.

^b^Motifs in intronic ORFs were classified by BLASTP provided by the NCBI website (https://blast.ncbi.nlm.nih.gov/). ND - Neither ORF longer than 100 amino acids nor motif was detected.

^c^Evolutionary origins of individual introns were inferred by combining phylogenetic analyses of HEs and intron position.

^d^HE can be overlapped with upstream exon.

^e^HE contains two LAGLIDADG motifs.

^f^The origins of the introns were not assessed, as no intronic ORF was found.

**Figure 2:**
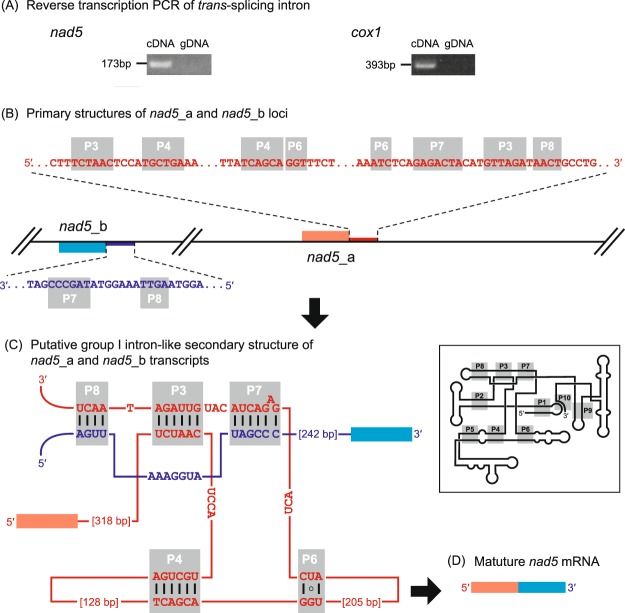
*Trans*-splicing for *nad5* and *cox1* gene expressions in the *Marophrys* mitochondrial genome. (**A**) Reverse transcription PCR using a set of primers specific to *nad5*_a and *nad5*_b loci (left) and that specific to *cox1*_a and *cox1*_b loci (right). The DNA fragment was amplified from the cDNA template which most likely contained the spliced product connecting the two RNA fragments transcribed from the two separate loci together (lanes labelled with “cDNA”). On the other hand, no specific amplification was observed in the PCR using the genomic DNA template due to the configuration of the two separate loci in the mtDNA (lanes labelled with “gDNA”). (**B-C**) Model for *nad5* mRNA *trans*-splicing. (**B**) Primary structures of *nad5*_a and *nad5*_b loci. mtDNA, exons, and introns are shown in thin black lines, boxed, and thick lines, respectively. The 5′ exon and subsequent intronic region are colored in red, while the 3′ exon and its preceding intronic region are indicated in blue. The two loci are located on the different strands, and thus transcribed independently from each other. (**C**) Putative group I intron-like secondary structure of *nad5*_a and *nad5*_b transcripts. Five stem-loop structures conserved among group I intron ribozymes (P3, P4, P6, P7, and P8) can be formed within the *nad5*_a transcript and between the *nad5*_a and *nad5*_b transcripts. This secondary structure was predicted by RNAweasel followed by manual inspection and modification. Watson–Crick base pairings and a wobble bond in the five stem-loop structures are indicated by the black lines and a circle, respectively. The typical secondary structure of group I intron ribozymes is schematically shown as an inset. (**D**) Mature *nad5* mRNA.

The gene for NADH dehydrogenase subunit 5 (*nad5*) appeared to be split into two distant loci in the mtDNA, which were designated as *nad5*_a and *nad5*_b encoding the N- and C-terminal halves of the protein, respectively (Fig. 1). According to the organization of the two loci in the mtDNA, they are most likely transcribed independently. We successfully obtained the evidence for a single, contentious mRNA molecule comprising the transcripts from *nad5*_a and *nad5*_b by performing reverse transcription (RT)-PCR using cDNA as template and two primers—one specific to the *nad5*_a nucleotide sequence and the other to the *nad5*_b nucleotide sequence (Figs 2A and S1B). Thus, the expression of *nad5* most likely requires *trans*-splicing. Likewise, we identified separate loci encoding the N- and C-terminal of Cox1, termed *cox1*_a and *cox1*_b, respectively (Fig. 1). Again, the RT-PCR provided evidence for the transcripts from *cox1*_a and *cox1*_b being *trans*-spliced into a single mRNA molecule encoding the entire Cox1 (Figs 2A and S1B).

RNAweasel suggested that the 3′ and 5′ flanking region of the transcript from *nad5*_a and *nad5*_b loci, respectively, can form the group I-specific secondary structure together (Fig. 2B–D). Interestingly, two of the predicted stem structures, P7 and P8, can be folded by a combination of the transcripts from *nad5*_a and *nad5*_b loci (Fig. 2C). Therefore, the *trans*-splicing between *nad5*_a and *nad5*_b transcripts is most likely mediated by a group I intron (Fig. 2B–D). Prior to this study, the group I intron-mediated *trans*-splicing was found in the mtDNAs of a placozoan^43^, green algae^44,45^ and an arbuscular mycorrhizal fungus^46^. However, these examples were limited to the *cox1* and *rnl* genes. Hence, the *Marophrys* mtDNA is the first example of group I intron-mediated *trans*-splicing in *nad5*.

Neither RNAweasel nor Infernal detected any conserved motif of group I (or group II) introns in the nucleotide sequences flanking the *cox1*_a or *cox1*_b locus. Thus, we currently have no insight into the mechanism mediating the *trans*-splicing between the transcripts from the two *cox1* loci.

### Origins of group I introns harboring HEs

Overall, 11 of the 19 introns identified in the *Marophrys* mtDNA harbor intronic ORFs encoding LAGLIDADG motif-containing HEs, which have been typically been found in group I introns^16^. All of the HEs in the *Marophrys* mtDNA belong to either LAGLIDADG_1 (pfam00961) or LAGLIDADG_2 (pfam031611). Here, we explored the evolutionary origins of the 11 group I introns harboring HEs by combining phylogenetic affinities of the HEs and the insertion positions of the introns. We are aware of the cases in which the evolution of a group I intron and that of the corresponding intron-encoded protein disagreed to one another^47^. Unfortunately, we could not exam whether introns and their intron-encoded proteins coevolved, as the intron (nucleotide) sequences dealt in this study are too diverged for phylogenetic analyses. Thus, we assumed the coevolution of each pair of a group I introns and its HE in the following sections. Table 1 summarizes the group I introns found in the *Marophyrs* mtDNA and their putative origins.

#### Introns sharing the origins with green algal organellar genomes

**Figure 3:**
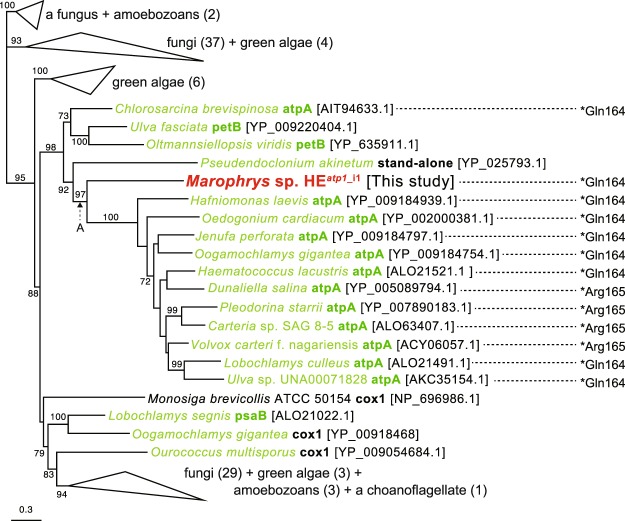
Maximum likelihood (ML) analysis of the homing endonucleases harbored in the first intron in *atp1* (HE^*atp1*_i1^) in the *Marophrys* mitochondrial genome. OTU names consist of species name, intron-hosting gene (bold) and accession number in brackets. Green algal sequences are shown in green. Gene names are colored in green when they reside in plastid genomes. The inserted positions of *atpA*/*atp1* introns are presented the numbers of the amino acid residues correspond to the *Marophrys* sequence. Asterisks indicate insertion site in triplet codon. For instance, “*Gln164” means that an intron was found between the codon for Gln^164^ and that for the 163^th^ amino acid (phase class = 0). Ultrafast bootstrap values higher than 70% are shown. Collapsed clades are indicated by triangles and the linages included in the clade are shown with the number of genes in parentheses. The detailed ML tree is given in Fig. S2.

*Marophrys atp1* intron (*atp1*_i1) harbors an HE (HE^*atp1_*i1^). The HE phylogeny grouped HE^*atp1_*i1^ with those harbored in *atpA* introns, which were found in the plastid genomes (plDNAs) of green algae belonging to core Chlorophyta, with an ML bootstrap value (MLBP) of 97% (node A in Figs 3 and S2). The *atpA* introns in green algal plDNA and *Marophrys atp1*_i1 appeared to be inserted in the homologous positions, namely, phase 0 of the 164^th^ codon for glutamine in *atp1* and the 165^th^ codon for arginine in *atpA* (Figs 3 and S2). The results described above consistently and strongly suggest that lateral transfer of an intron took place between a centrohelid mtDNA and a green algal plDNA. Nevertheless, the data presented above are insufficient to draw definitive conclusions about whether the ancestral intron emerged in an mtDNA or a plDNA. Interestingly, DNA transfer from an mtDNA to a plDNA has been considered to occur rarely^48,49^, and many cases of DNA transfer with the opposite direction have been documented^50,51^. Thus, we favor the intron transfer from a green alga to a centrohelid over that in the opposite direction. Moreover, centrohelids probably encounter opportunities to uptake the genetic materials of green algae in the natural environments. *Marophrys* sp. SRT127 and other centrohelids prey on green algae, and some centrohelids are capable of sequestering the plastids of their prey algae for a certain period, which is known as kleptoplasty^52^. Taking these findings together, we propose that *atp1*_i1 in the *Marophrys* mtDNA was laterally transferred from a green algal plDNA.

We also detected four *rnl* introns in *Marophrys* mtDNA that shared origins with those in organellar genomes (mtDNAs or plDNAs) in green algae. The HE harbored in *Marophrys rnl*_i3 (HE^*rnl*_i3^) grouped together with the HEs in two mitochondrial and one plastid *rnl* genes in green algae with an MLBP of 97% (node A in Fig. S3). Among the introns harboring the HEs united by node A in Fig. S3, *Marophrys rnl*_i3 and two other introns appeared to be inserted in the homologous position (i.e., the introns were found between G^1809^ and A^1810^; nucleotide numbering is based on the *Marophrys rnl* gene).

The HE harbored in *Marophrys rnl*_i4 (HE^*rnl*_i4^) branched at the base of the clade of nucleus-encoded HEs in land plants with an MLBP of 87% (node A in Fig. S4). The nucleus-encoded HEs in land plants are not encoded by intronic ORFs (designated as “stand-alone” in Fig. S4). *Marophrys* HE^*rnl*_i4^ and the nucleus-encoded HEs were then connected with the HEs harbored in eight plastid and one mitochondrial *rnl* introns in green algae with an MLBP of 85% (node B in Fig. S4). In the clade united by node B, HE^*rnl*_i4^ was excluded from both stand-alone HEs in land plants, and HEs in organellar *rnl* introns in green algae. It is difficult to clarify the origin of HE^*rnl*_i4^ with a confidence based on the HE phylogeny (Fig. S4). However, the particular HE in the *Marophrys* mtDNA is encoded by an intronic ORF (not by a stand-alone), and *Marophrys rnl*_i4 and eight plastid *rnl* introns in green algae appeared to be inserted between T^2151^ and C^2152^ (nucleotide numbering is based on the *Marophrys rnl* gene). Taking these findings together, we propose that *Marophrys rnl*_i4 and the eight *rnl* introns in green algal plDNA are derived from a single *rnl* intron.

The HE harbored in *Marophrys rnl*_i6 (HE^*rnl*_i6^) and those found in four plastid and four mitochondrial *rnl* introns in green algae formed a clade with an MLBP of 91% (node C in Fig. S4). Among the introns harboring the HEs united by node C in Fig. S4, the *Marophrys* intron was found to share the insertion position with three plastid introns (i.e., the introns were found between C^2394^ and A^2395^; nucleotide numbering is based on the *Marophrys rnl* gene). We concluded that *Marophrys rnl*_i6 and the *rnl* introns found in green algal organellar genomes share the common ancestor.

The HE harbored in *Marophrys rnl*_i8 (HE^*rnl*_i8^) and that in the *rnl* intron in the *Acanthamoeba castellanii* mtDNA grouped together with an MLBP of 94%, and the two introns appeared to be inserted between G^2482^ and A^2483^ (nucleotide numbering is based on the *Marophrys rnl* gene; node A in Fig. S5). Moreover, the clade of HE^*rnl*_i8^ and the *Acanthamoeba* HE clustered with two stand-alone HEs in bacterial genomes, HEs found in 24 *rnl* introns in green algal mtDNAs/plDNAs and a single *rnl* intron in a diatom mtDNA (MLBP = 86%, node B in Fig. S5). The vast majority of the *rnl* introns described here were found in a homologous position. Altogether, we suspect that the ancestral intron, which gave rise to the ones in the *Marophrys* and *Acanthamoeba* mt *rnl* genes, resided in the *rnl* gene in a green algal mtDNA or plDNA.

By combining the phylogenetic affinities of HEs and intron positions, we propose that the four *rnl* HEs (and their host introns) found in the *Marophrys* mtDNA share origins with those in organellar genomes in green algae as well as those of *atp1*. The *atp1* and *rnl* introns described above were likely transmitted from green algae to *Marophrys* by considering the predator–prey relationship between centrohelids and green algae in the wild (see above). We presume that, once mobile genetic elements resided in a centrohelid mtDNA, these elements may have persisted in the descendant genomes, as centrohelids are unicellular and asexual eukaryotic lineage^53^. Unfortunately, the data obtained in this study failed to (i) pinpoint the green algal species that donated the five introns or (ii) clarify whether mtDNA or plDNA was the origin of each of the four *rnl* introns. It is also important to re-examine the putative green algal origins of the five introns (and their HEs) in future studies incorporating organellar genome data sampled from much broader eukaryotes (green algae and centrohelids in particular).

It should be noted that, in the strict sense, we cannot rule out the possibility of intron-HE coevolution being violated in the *Marophyrs* mtDNA, as the intron phylogenies were not examined due to their extremely divergent natures. Nevertheless, the HE phylogenies (Figs 3 and S2–5) firmly suggest that the HE-coding DNA fragments were transferred from green algal organellar genomes to centrohelid mtDNAs.

#### Other introns

The HE found in *Marophrys rns*_i1 was placed within a radiation of the HEs in *rns* genes in green algal plastid and bacterial genomes, and this clade received an MLBP of 96% (node D in Fig. S4). Within this clade, the affinity between the *Marophrys* and bacterial HEs could not be excluded, leaving the origin of this intron inconclusive.

The HE phylogeny tied together the HE found in *Marophrys cob*_i2 and that in a fungal *cob* intron with an MLBP of 86% (node E in Fig. S4). However, the inserted positions of the two *cob* introns appeared to be distant from each other. Thus, it remains unclear whether the two *cob* introns genuinely share the same origin.

The HE phylogeny united the HE found in *Marophrys cox1*_i1 (HE^*cox1*_i1^) and those in two green algal and three fungal *cox1* genes with an MLBP of 78% (node A in Fig. S6). However, HE^*cox1*_i1^ showed no special affinity to any of the five HEs in this clade. The inserted positions of *Marophrys cox1*_i1 and those harboring the five HEs grouped with HE^*cox1*_i1^ appeared to vary. We consider that the results described above are insufficient to infer the origin of *Marophrys cox1*_i1 with confidence.

As we yielded no insight for the origin of *cox1*_i3, *cox1*_i6 or *rnl*_i5, as none of their HEs showed any particular affinities to other HEs considered in the analyses (Figs S4 and S7).

### Plasmid and plasmid-derived genes

In addition to the circular mtDNA, we identified a linear plasmid of 5,877 bp in length with inverted repeats at both ends (Fig. S8). This plasmid contains genes encoding virus-type *dpo* and *rpo* (Fig. S8). The two genes in the *Marophrys* plasmid most likely use UGA for tryptophan codon instead of translation termination signal. Although no experimental evidence is available, we consider that the linear plasmid localizes in the *Marophrys* mitochondrion for two reasons described below. First, the structure and gene content of the *Marophrys* plasmid resemble those of mitochondrial plasmids found in land plants, fungi, amoeba and a ciliate *Oxytricha trifallax*^24–29^. Second, the circular mtDNA and linear plasmid share the same deviant genetic code. Given the patchy distribution in the tree of eukaryote, the linear plasmids are regarded as one of the mobile genetic elements^23,29,54^. Altogether, we here propose that the *Marophrys* plasmid was laterally acquired from an as-yet-unknown eukaryote.

The *Marophrys* plasmid showed clear similarity at the nucleotide level to the region containing *dpo* and *rpo* in the circular mtDNA (Fig. S8). As observed in other eukaryotes^25,54^, we suspect that the linear plasmid was integrated into the circular mtDNA in the *Marophrys* mitochondrion^55^. The inverted repeats were proposed to facilitate the linear plasmid to integrate into an mtDNA, and, in some cases, the entire plasmid including the repeats were found in the mtDNA^56^. In the *Marophrys* mitochondrion, the inverted repeats in the linear plasmid (102 bp in length) were found to be totally different from those surrounding *dpo* and *rpo* in the mtDNA (the regions highlighted by red arrows in Fig. 1; 7,659 bp), indicating that only two genes in the plasmid were integrated into the mtDNA. Nevertheless, it is attractive to hypothesize that plasmid integration (more precisely integration of *dpo* and *rpo*) triggered the duplication of a ~7.6 Kb region containing *nad9*, *cob*, *cox1*_a, two ORF and three tRNA genes observed in the current *Marophrys* mtDNA (red arrows in Fig. 1), as Sakurai *et al*.^57^ reported the structural change in an mtDNA led by plasmid integration. Unfortunately, the mtDNA from a single centrohelid species is insufficient to examine whether the inverted repeats in the *Marophrys* mtDNA arose with the plasmid integration. Relevant to the above issue, we need to clarify the mechanism by which plasmid integration introduced the inverted repeats in the recipient DNA molecule. As the first step toward resolving the issues mentioned above, we require additional mtDNA data from multiple centrohelids, particularly close relatives of *Marophrys*.

## Methods

### Isolation and culturing

A clonal culture of a centrohelid (strain SRT127) was established using micropipetting method from seawater sample that had been collected from Tokyo Bay, Tokyo, Japan (35.6281°N, 139.7713°E), on July 30, 2011. Based on the morphological characteristics, strain SRT127 was identified as a member of the genus *Marophyrs* (Fig. S9).

*Marophrys* sp. strain SRT127 was maintained in MNK medium (http://mcc.nies.go.jp/02medium.html#mnk) with a green alga *Pyramimonas* sp. as prey at 20 °C under a 14-h light / 10-h dark cycle until it died in March 2016.

### DNA/RNA extraction and cDNA synthesis

The centrohelid cells were collected by centrifugation once they had reached confluence and the algal cells were hardly observed in the culture medium under a light microscope. Genomic DNA was extracted with 25:24:1 of phenol:chloroform:isoamyl alcohol and purified by ethanol precipitation. Total RNA was extracted using TRIzol (Thermo Fisher Scientific), following manufacturer’s instructions. The extracted RNA was used to synthesize random hexamer-primed cDNA with SuperScript II reverse transcriptase (Thermo Fisher Scientific). The cDNA was used for the PCR experiments to confirm intron splicing (see detail in *Genome annotation* section).

### Sequencing of the *Marophrys* sp. mitochondrial genome

Approximately 10 μg of the extracted DNA was submitted to Illumina Sequencing technology (HiSeq 2000 at Eurofins Genomics). A total of 125,733,479 paired-end reads of 100 bp were generated (Approx. 25 Gb in total) and assembled by SPAdes^58^. To identify mitochondrial sequences from the assembly data, a TBLASTN^59^ search was performed using the putative amino acid sequences of mitochondrion-encoded proteins in a jakobid *Andalucia godoyi*^4^ as queries. A total of 228 contigs were recovered as candidates for *Marophrys* mtDNA fragments at the threshold *E*-value of <1e−10. Then, the candidate contigs were used as a queries for a BLASTN^59^ search against the NCBI non-redundant nucleotides database. The contigs hit to bacterial sequences with ≥ 95% nucleotide identity in the second BLAST analysis were discarded as the genome fragments originated from bacteria contaminating in the *Marophrys* culture. After filtering bacterial sequences, two contigs, which were 90,947 and 7,652 bp in length, were recovered as mtDNA fragments of *Marophrys*. By mapping the paired-end reads on the two putative mtDNA fragments using Bowtie2^60^, we additionally identified a contig of 6,811 bp in length as a mtDNA fragment. The third contig was overlooked by the first TBLASTN^59^ analysis, as this region only carries *dpo* and *rpo* genes that encode non-typical mitochondrion-encoded proteins. The physical continuity of three candidate mtDNA fragments was confirmed by PCR. Finally, the *Marophrys* mtDNA was reconstructed as a circular molecule of 113,062 bp in length.

### Linear plasmid carrying *dpo* and *rpo*

A contig of 5,877 bp in length was found by a TBLASTN^59^ search against the assembly data using the *dpo* and *rpo* genes identified in the circular-mapped mtDNA as queries. This 5,877 bp-contig appeared to bear inverted repeats of 102 bp in length at both 5′ and 3′ ends, and to carry only *dpo* and *rpo*. To determine whether the contig is a linear or circular molecule, we mapped the paired-end reads to the contig using Bowtie2^60^. We also conducted a PCR experiment to connect both ends of the contig as done to the circular mtDNA (see above). As neither of the aforementioned experiments positively supported the circular structure of the contig (data not shown), we concluded that the 5,877-bp contig represents a linear DNA molecule.

### Genome annotation

The *Marophrys* mtDNA was annotated by Prokka^61^, followed by manual curation. NCBI translational Table 4 (https://www.ncbi.nlm.nih.gov/Taxonomy/Utils/wprintgc.cgi#SG4) was applied during the aforementioned annotation, because UGA codons are most likely assigned as tryptophan, not as the termination signal for translation in the *Marophrys* mtDNA. When a gene appeared to contain one or more introns, the corresponding cDNA was amplified by PCR with the primers listed in Table S2, and the amplicons (Fig. S1A,B) were sequenced by the Sanger method. The precise intron–exon boundaries were determined by comparing the corresponding cDNA and genome sequences. The type of introns was predicted by RNAweasel (http://megasun.bch.umontreal.ca/cgi-bin/RNAweasel/RNAweaselInterface.pl). The secondary structural motif conserved among group I introns was also searched by Infernal^62^ with covariance models built by Nawrocki *et al*.^63^.

### Phylogenetic analyses of HEs

The conceptual amino acid sequences of the HEs identified in the *Marophrys* mtDNA were used as queries for a BLASTP^59^ search against the NCBI nr database (*E*-values threshold < 1e−10) to generate phylogenetic datasets of HEs. The HE sequences retrieved from the NCBI nr database, which shared >90% identity at the amino acid sequence level, were clustered by CD-HIT^64,65^. To reduce the redundancy, the representative sequences from each cluster were selected to build the phylogenetic datasets analyzed in this study. The HE sequences harbored in the third and sixth introns of *cox1* gene appeared to share the same evolutionary origin, and we prepared a single dataset including both HEs and their related sequences. For a similar reason, we prepared a single dataset containing HEs harbored in three of the introns found in *rnl* gene, a single intron in *rns* gene and one of the introns found in *cob* gene in the *Marophrys* mtDNA. Individual datasets were subjected to MAFFT^66^ with the LINSI option for calculating the alignments. The ambiguously aligned sites in the alignments were discarded manually. Maximum likelihood (ML) analyses using IQ-TREE^67^ were conducted on each alignment with 1,000 ultrafast bootstrap replicates^68^. The substitution models were selected by ModelFinder^69^ implemented in IQ-TREE.

## Supplementary information

Supplementary Figures

## Data Availability

The *Marophrys* mtDNA sequences with annotation are available at GenBank/EMBL/DDBJ (accession nos AP019310 and AP019311). The alignments of HEs used to estimate phylogenetic tree are available from the corresponding author on request.
