# Supplementary material for: Horizontally-acquired genetic elements in the mitochondrial genome of a centrohelid Marophrys sp. SRT127
Source: Sci Rep. 2019 Mar 19;9:4850. doi: 10.1038/s41598-019-41238-6 (PMC6425028; doi:10.1038/s41598-019-41238-6)
Supplement: Supplementary file 1 — Supplementary Figures [file 41598_2019_41238_MOESM1_ESM.pdf]

Horizontally-acquired genetic elements in the mitochondrial genome of a centrohelid *Marophrys* sp. SRT127

Yuki Nishimura[1,a,*], Takashi Shiratori[1,b], Ken-ichiro Ishida[1], Tetsuo Hashimoto[1,2], Moriya Ohkuma[3], Yuji Inagaki[1,2]

[1] Graduate School of Life and Environmental Sciences, University of Tsukuba, Tsukuba, Japan.

[2] Center for Computational Sciences, University of Tsukuba, Tsukuba, Japan.

[3] RIKEN BioResource Research Center, Japan Collection of Microorganisms Microbe Division, Tsukuba, Japan.

[a]current address: RIKEN BioResource Research Center, Japan Collection of Microorganisms Microbe Division, Tsukuba, Japan.

[b]current address: Japan Agency for Marine-Earth Science and Technology (JAMSTEC), Yokosuka, Japan.

[*]Corresponding author: E-mail: yuki.nishimura@riken.jp

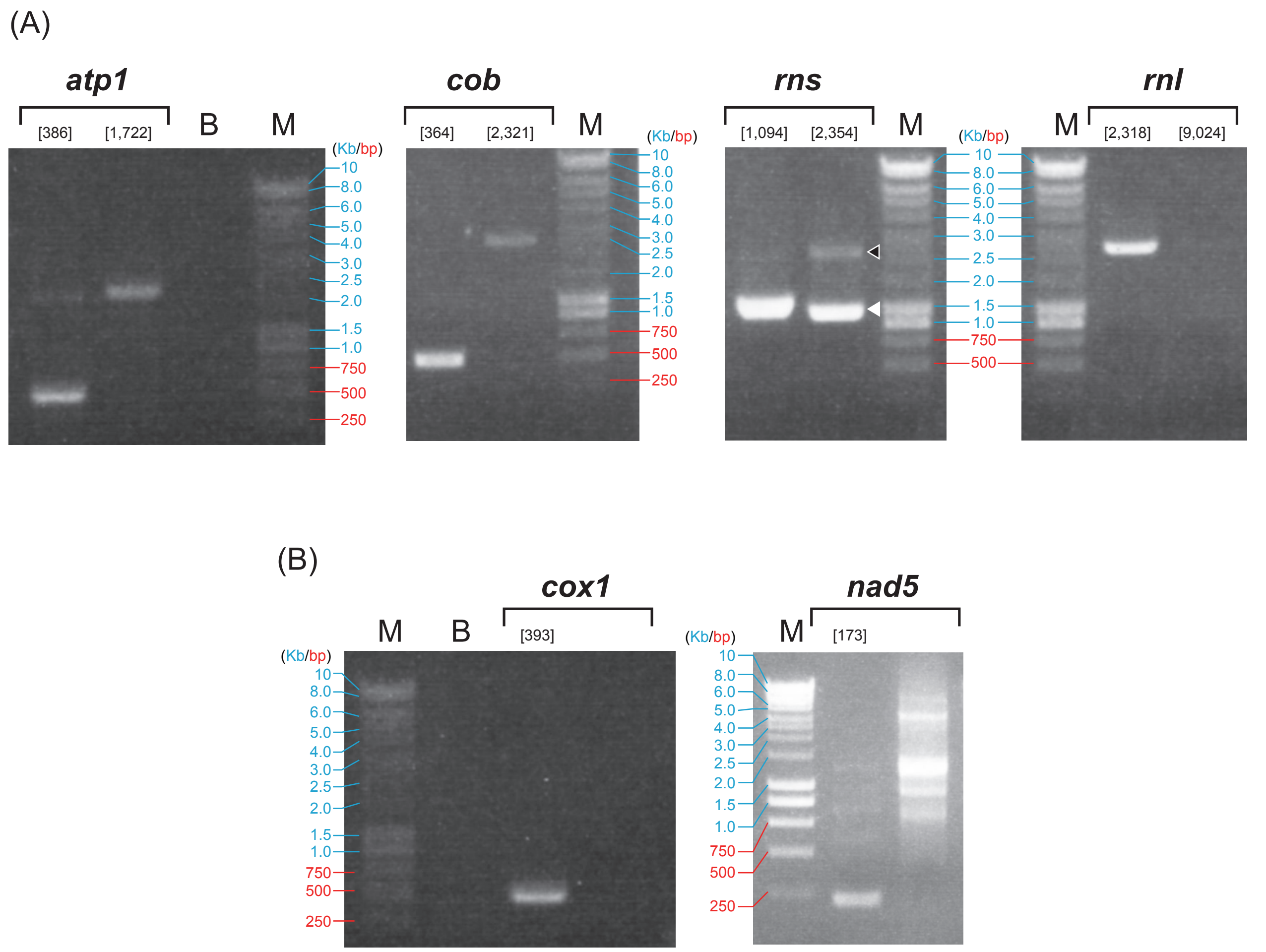

Supplementary Figure 1. PCR experiments assessing intron splicing. (A) Experiments on *atp1*, *cob*, *rns* and *rnl*. For each of the four genes, we prepared a set of primers that matches the *Marophrys* mtDNA sequence exactly and encompasses an intron/introns (see Table S2 for the detail). The results from the PCR using the cDNA sample and those from the genomic DNA (gDNA) sample were shown in the left and right lanes, respectively. The expected lengths of the PCR product are shown in the brackets. Notes—1) the primers for *rns* amplified two DNA fragments from the gDNA sample, one is of *Marophrys* (containing an intron, 1,722 bp; highlighted by an open arrowhead), and the other is of a green alga *Pyramimonas* sp. that was fed to the centrohelid in the laboratory culture (924 bp; highlighted by a filled arrowhead). 2) The large *rnl* gene fragment containing 8 introns (9,024 bp) was failed to be amplified from the gDNA sample. (B) Experiments on *cox1* and *nad5*. For each of the two genes, we prepared a set of primers that are specific to the two separate loci (*cox1*_a/*nad5*_a and *cox1*_b/*nad5*_b; see Fig. 1). In theory, the DNA amplification bridging the two separate loci occurred only in the PCR using the cDNA sample. The expected lengths of the PCR product amplified from the cDNA sample are shown in the brackets. As no primer-specific amplification was expected for the experiments using the gDNA template, no expected size was provided. We observed the DNA amplification from the gDNA using the primers for *nad5*, albeit they were most likely of non-specific. Blank lanes are labelled as “B”. Lanes labelled by “M” indicate size markers for double-strand DNA.

Supplementary Figure 2

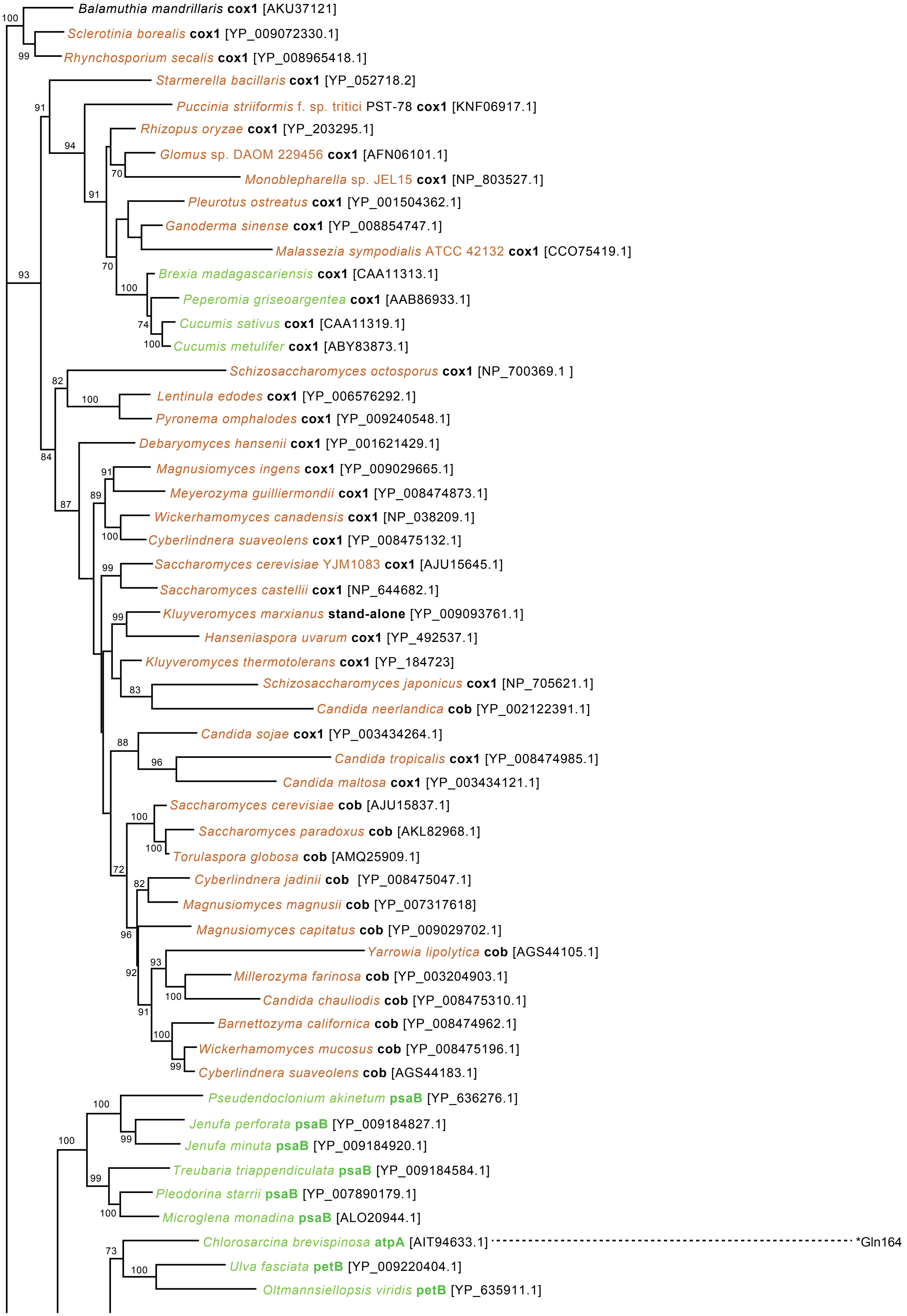

Supplementary Figure 2 (continued)

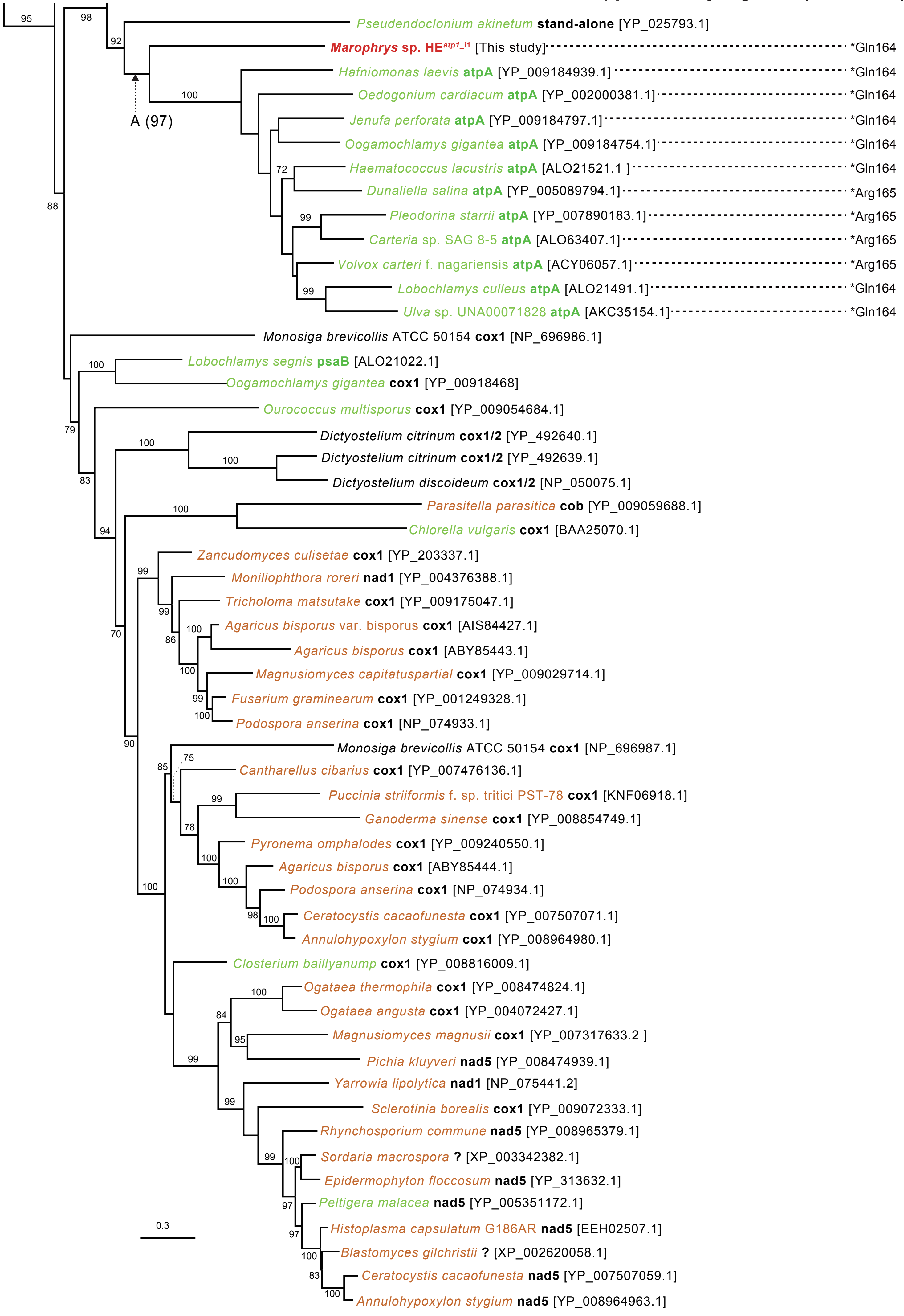

Supplementary Figure 2. Unrooted maximum-likelihood (ML) tree of homing endonuclease (HE) sequences including HE^{atp1_i1}. A phylogenetic analysis was performed on 117 sequences with 214 amino acids positions under the WAG + F + I + G4 substitution model. Each OTU names consists of species name, intron hosting gene (bold), and GenBank accession number surrounded by brackets. Green algal and fungal species names are colored in green and brown, respectively. Intron-hosting gene names are colored in green of plastid-encoded. For each *atp1*/*atpA* intron, we provied the number and amino acid identity of the intron-containing codon, which is based on the *Marophrys atp1* gene. Asterisks indicate the pahse classes of intron-inserted positions. Ultrafast bootstrap values are shown when they are greater than 70%. The key node to infer the origin of the intron in the *Marophrys atp1* gene (*atp1*_i1) and its HE (HE^{atp1_i1}) is labelled with “A” and the corresponding bootstrap value is shown in parentheses.

Supplementary Figure 3

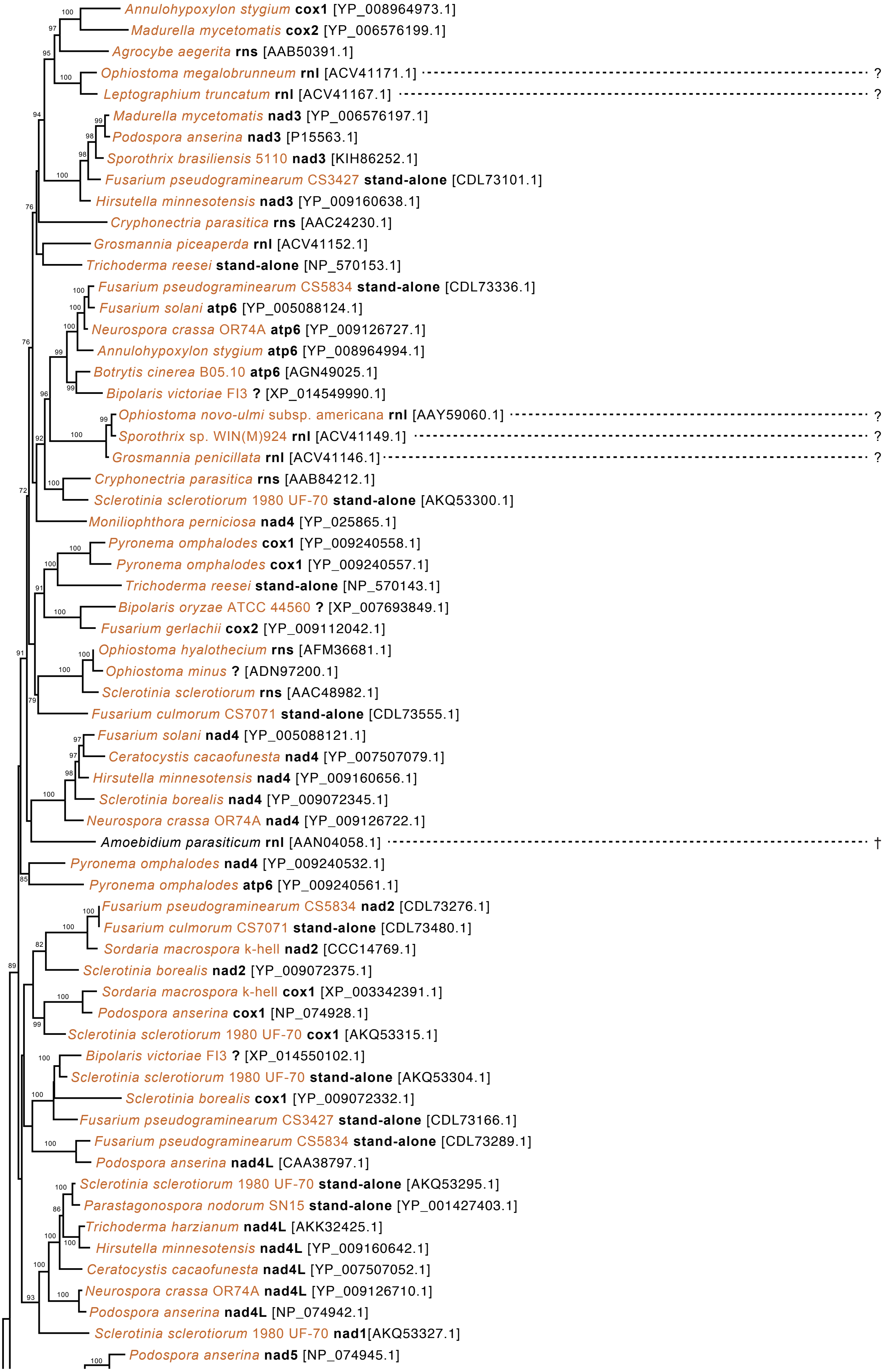

Supplementary Figure S3 (continued)

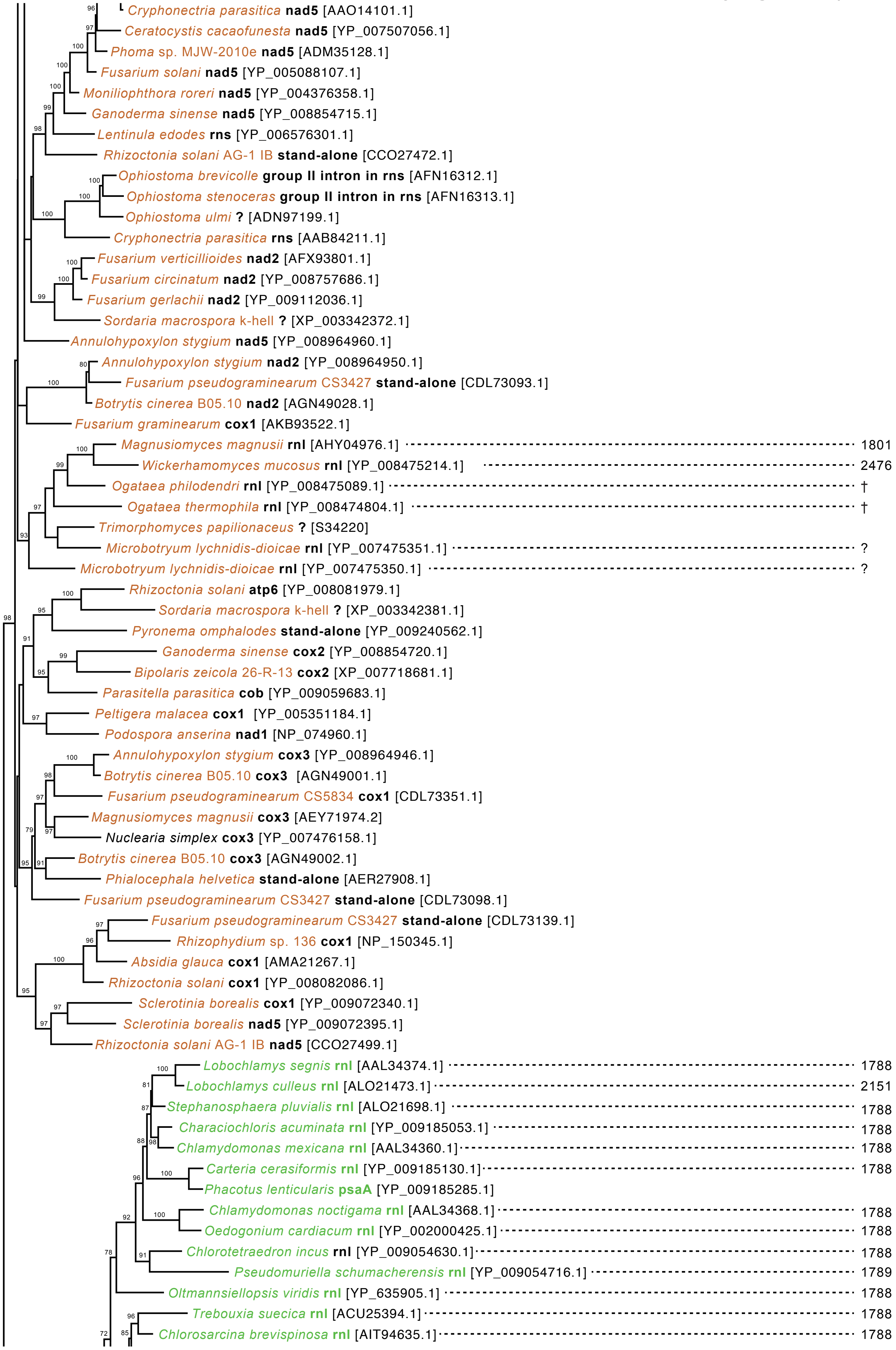

Supplementary Figure 3 (continued)

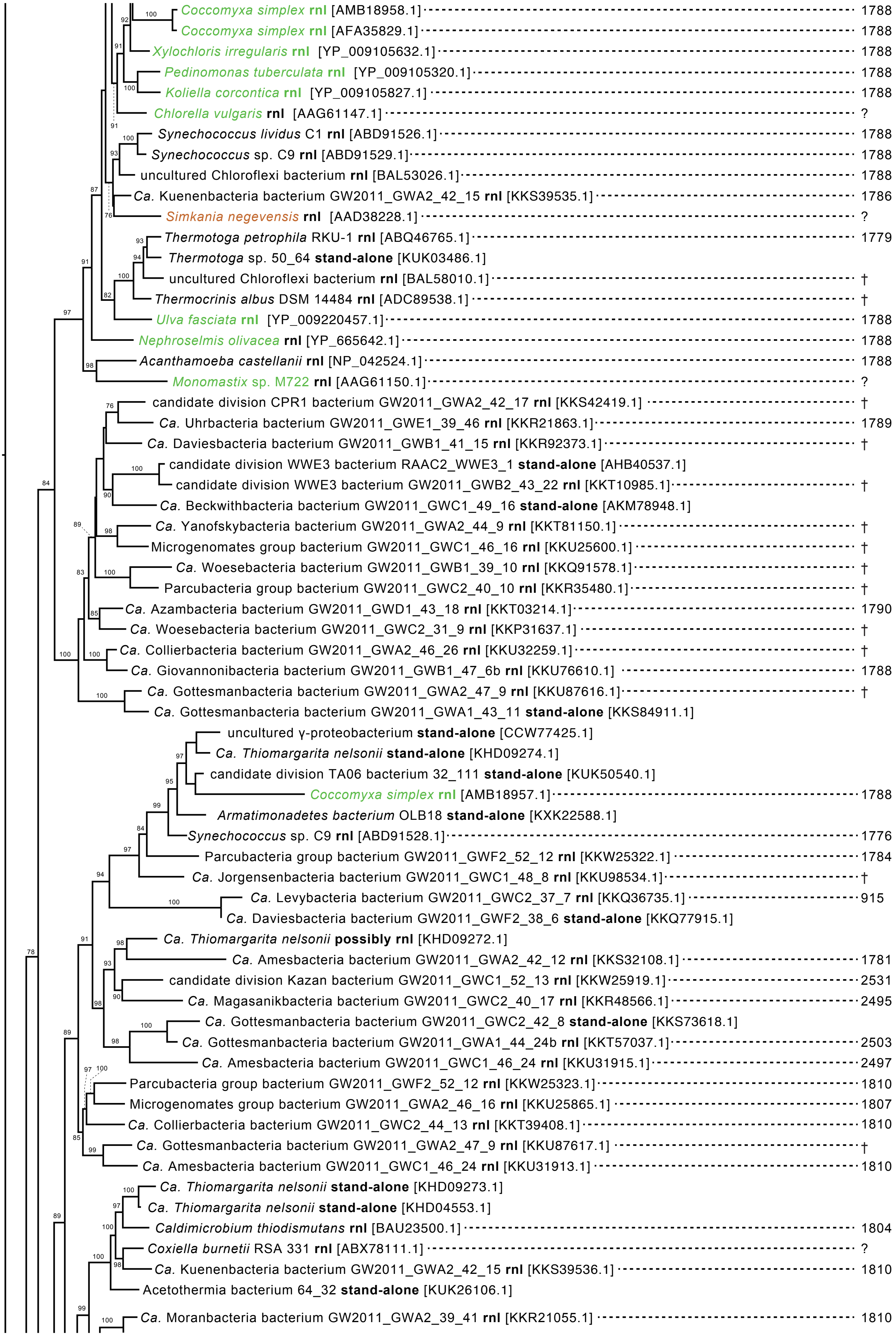

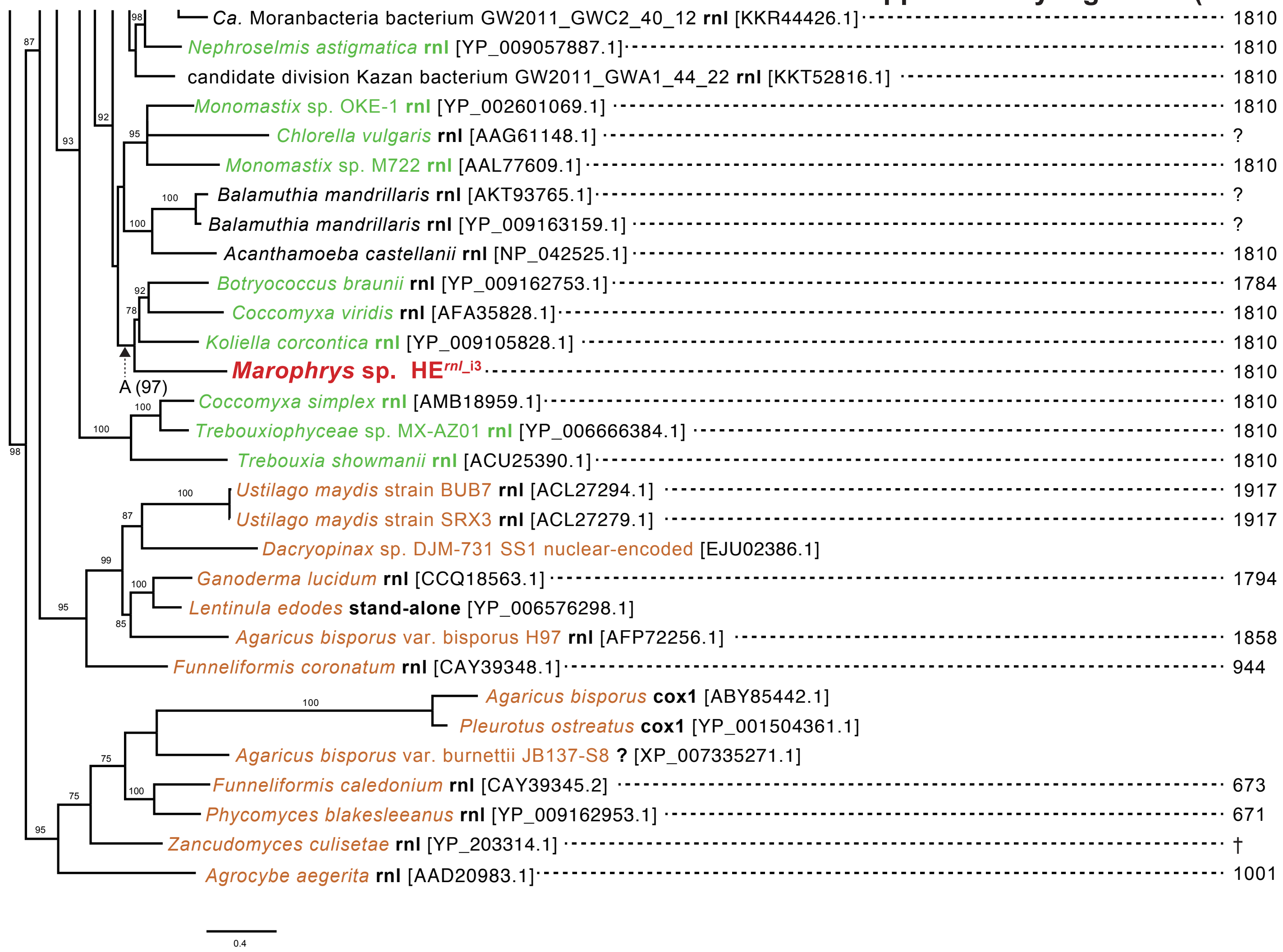

Supplementary Figure 3. Unrooted maximum-likelihood (ML) tree of homing endonuclease (HE) sequences including HE^{rnl_i3}. A phylogenetic analysis was performed on 119 sequences with 224 amino acid positions under the WAG + F + I + G4 substitution model. The key node to infer the originin of the third intron in *Marophrys rnl* (*rnl*_i3) and its HE (HE^{rnl_i3}) is labelled with "A" and the corresponding bootstrap value is shown in parentheses. For each *rnl* intron, we provided the number of the nucleotide that follow the particular intron immediately (the nucleotide numbering is based on the *Marophrys rnl* gene). Daggers indicate that the intron-inserted region is not conserved in the *Marophrys rnl* gene. Question marks mean that the genes hosting introns or intron-inserted positions are not recorded in the NCBI database. Other details are the same as in the legend for Figure S2.

Supplementary Figure 4

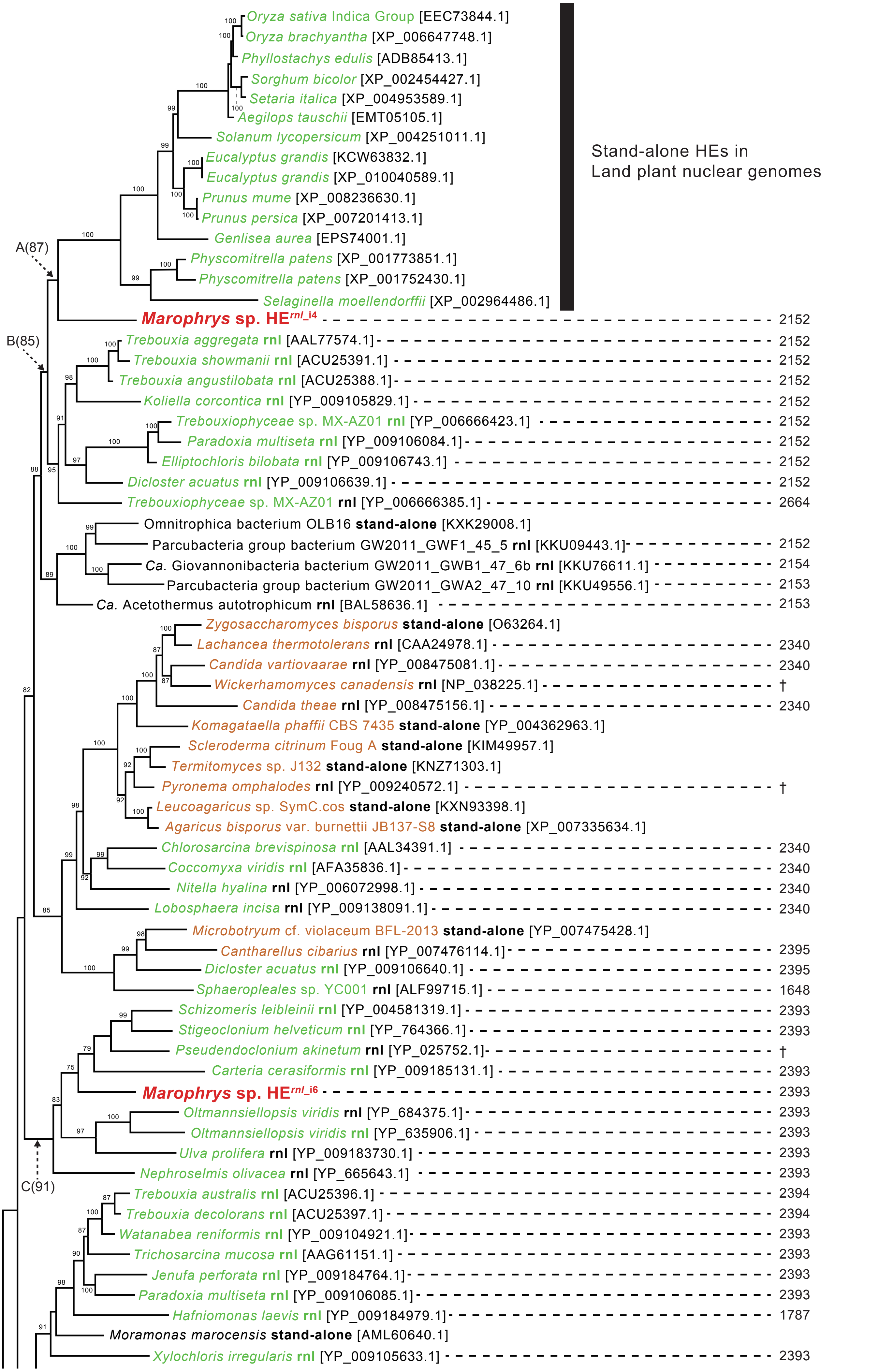

Supplementary Figure 4 (continued)

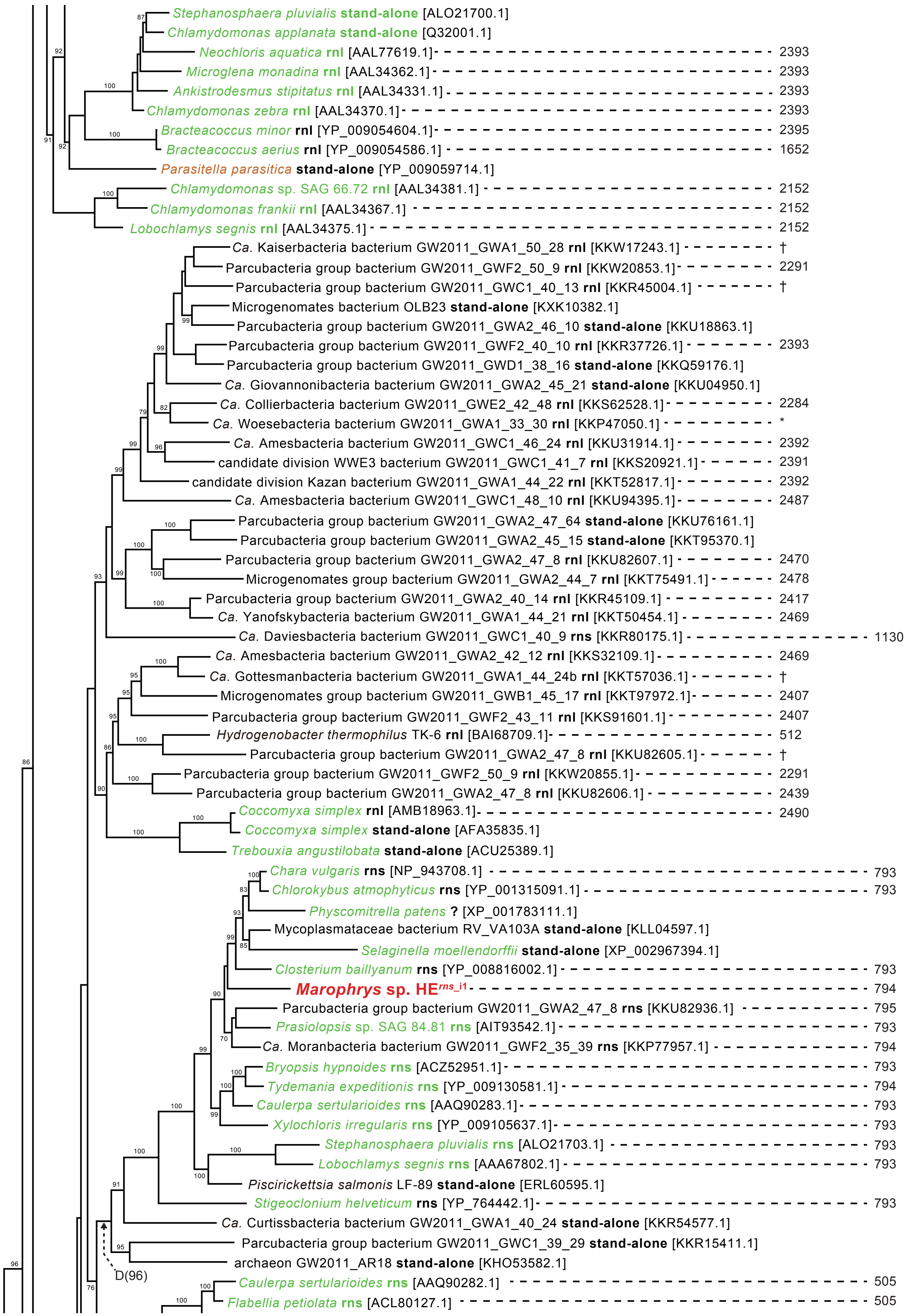

Supplementary Figure 4 (continued)

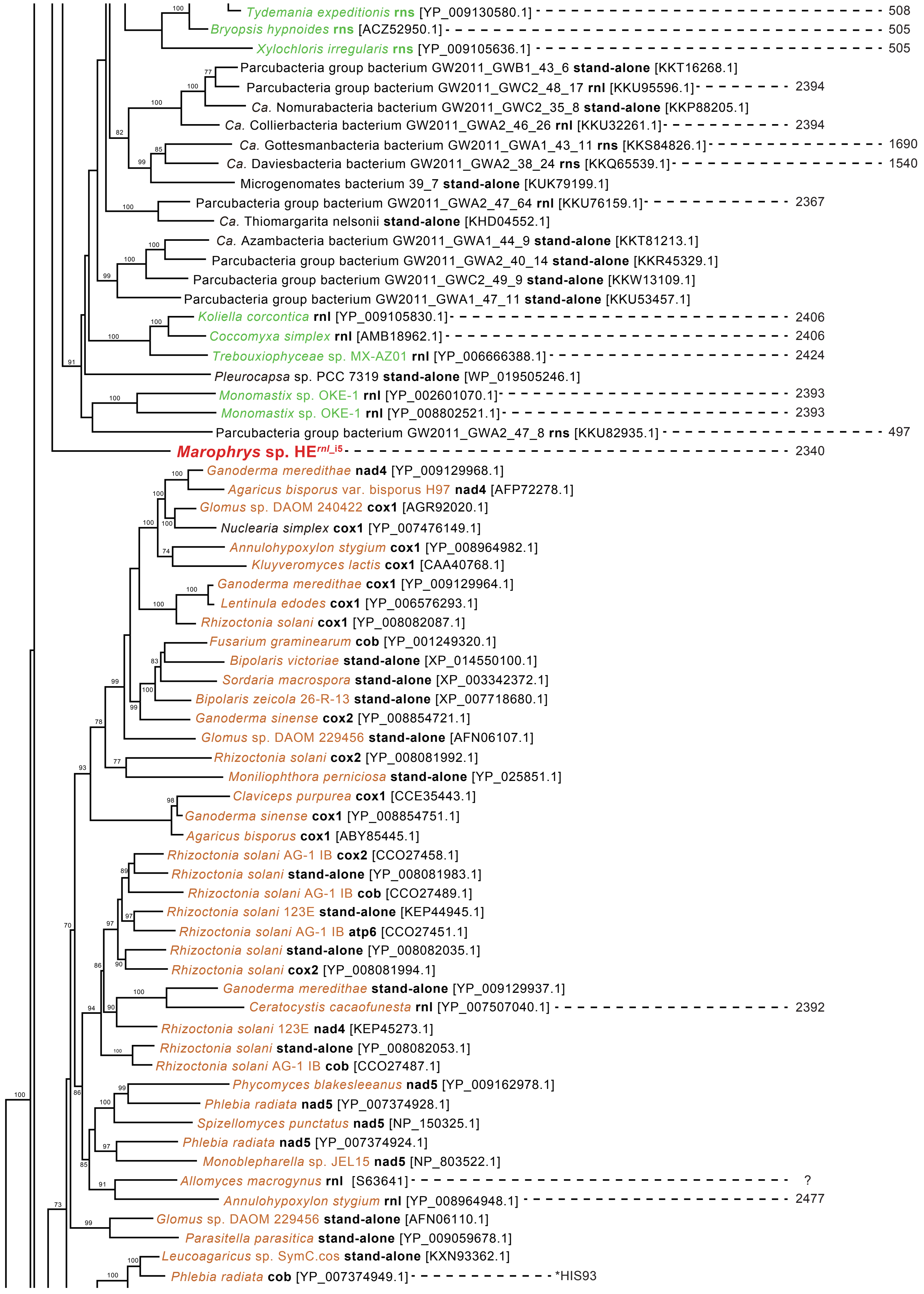

Supplementary Figure 4 (continued)

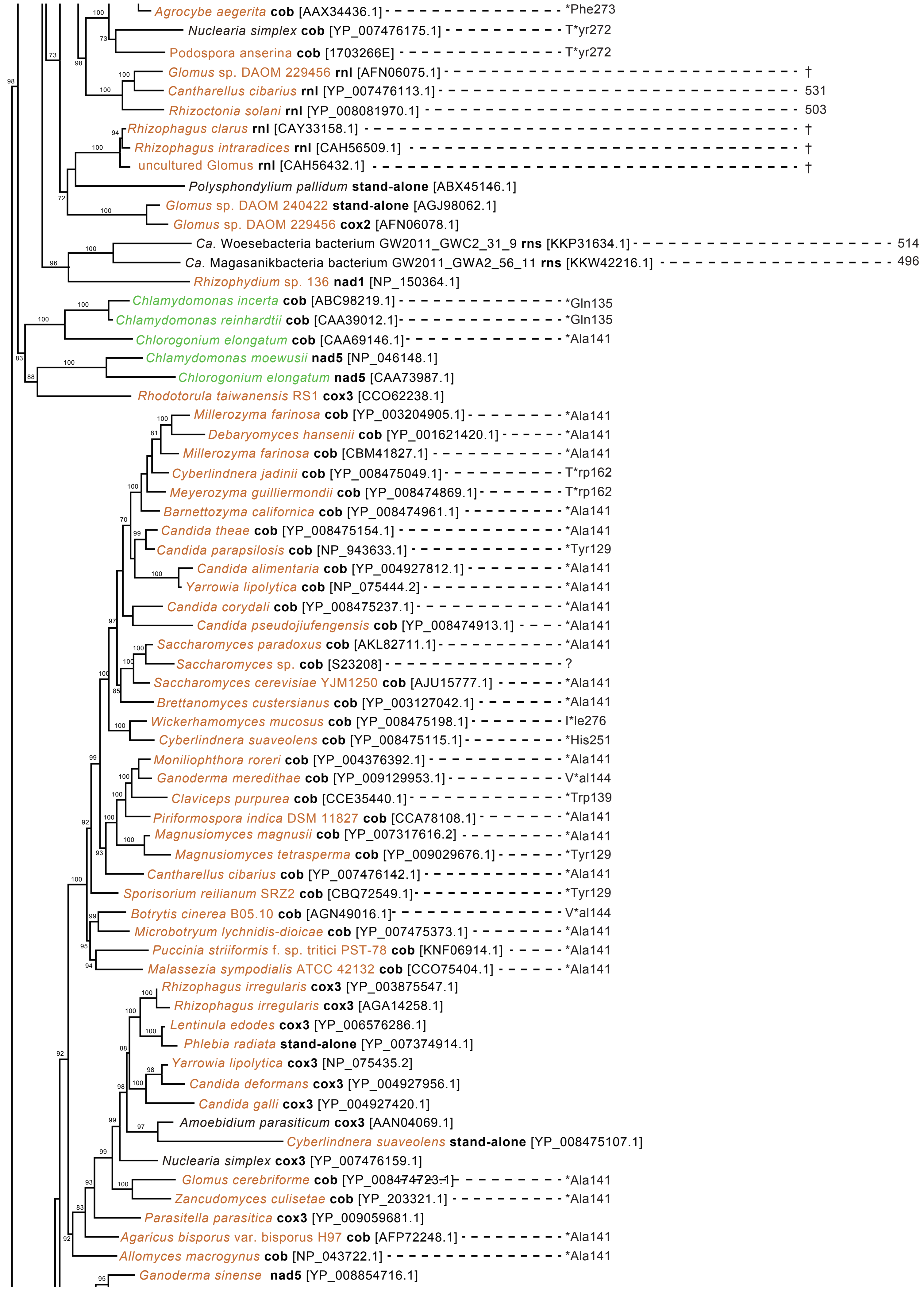

Supplementary Figure 4 (continued)

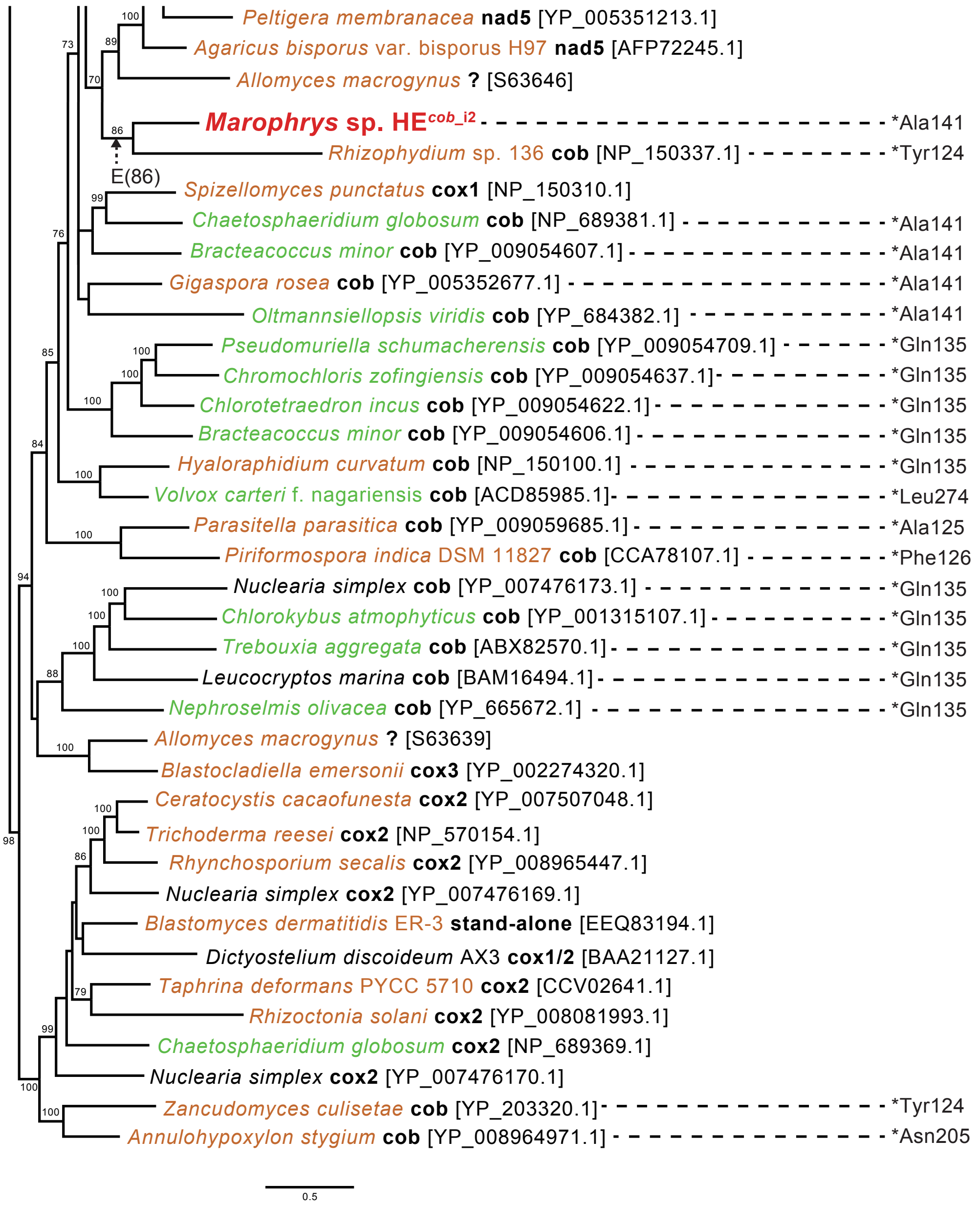

Supplementary Figure 4. Unrooted maximum-likelihood (ML) tree of LADLIDADG_2 type homing endonuclease (HE) sequences. A phylogenetic analysis was performed on 305 sequences with 165 amino acid positions under the LG + F + G4 substitution model. The key nodes to infer the origin of the forth intron in the *Marophrys rnl* (*rnl*_i4) and its HE (HE^{rnl_i4}) are labelled with "A" and "B". Likewise, the nodes labelled with "C", "D" and "E" are important to infer the origin of sixth intron in the *Marophrys rnl* gene (*rnl*_i6) and its HE (HE^{rnl_i6}), that of the first intron in *rns* gene (*rns*_i1) and its HE (HE^{rns_i1}) and that of the second intron in *cob* gene (*cob*_i2) and its HE (HE^{cob_i2}), respectively. The bootstrap values for nodes A-E are shown in parentheses. Other details are the same as in the legend for Figs. S2-S3.

Supplementary Figure 5

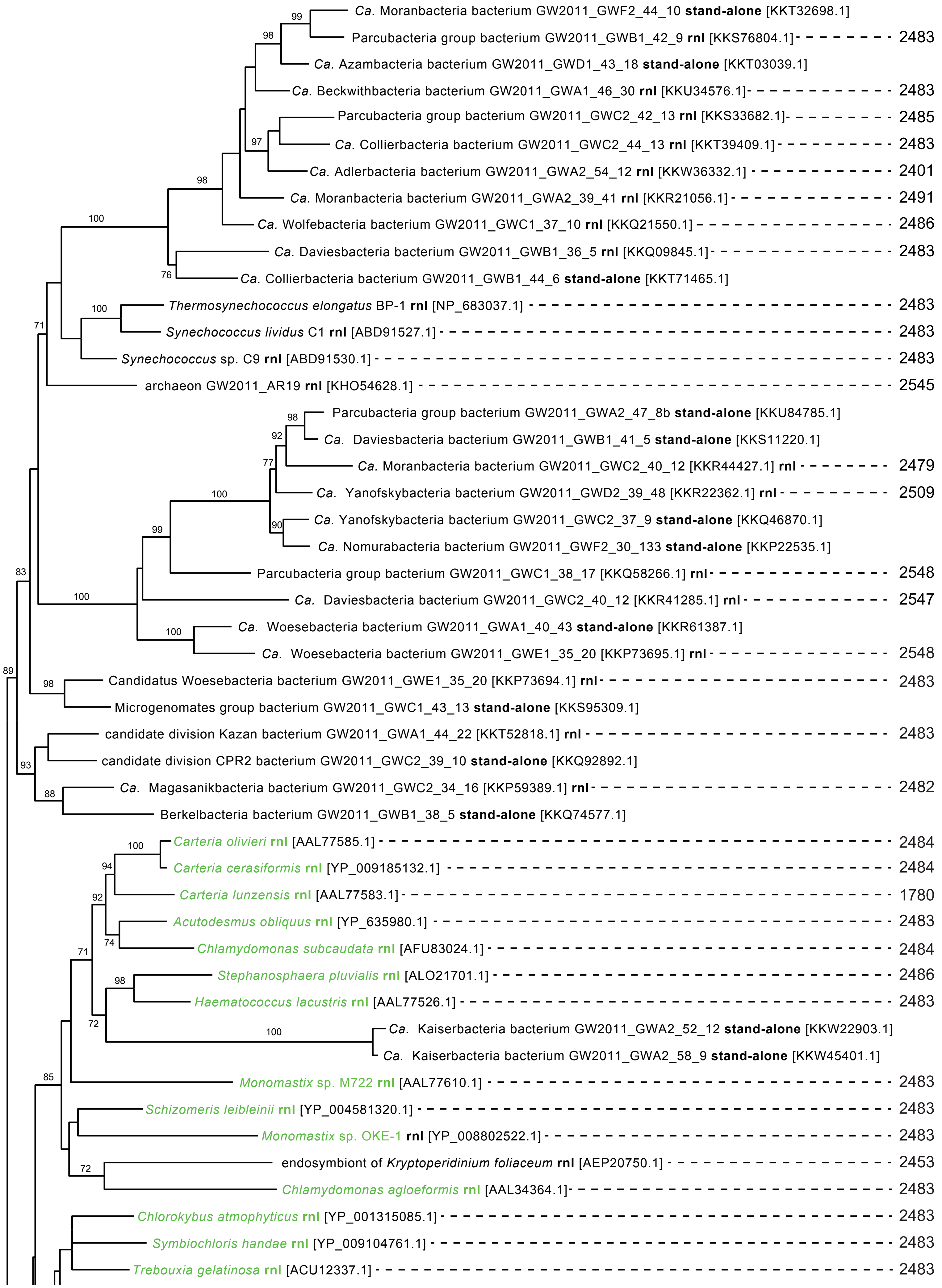

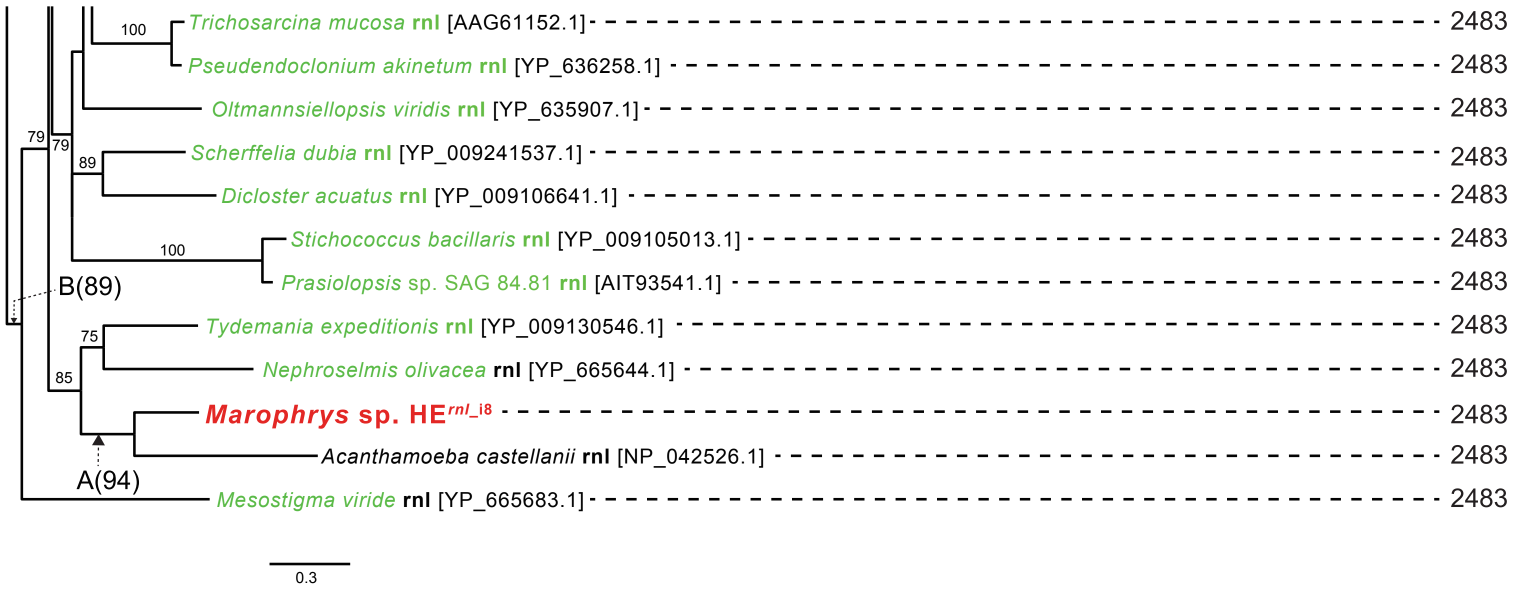

Supplementary Figure 5. Unrooted maximum-likelihood (ML) tree of homing endonuclease (HE) sequences including HE^{rnl_i8}. A phylogenetic analysis was performed on 60 sequences with 143 amino acid positions under the LG + F + I + G4 substitution model. The key nodes to infer the origin of the eight intron in *Marophrys rnl* gene (*rnl*_i8) and its HE (HE^{rnl_i8}) are labelled with “A” and “B” , and the corresponding bootstrap values are shown in parentheses. Other details are the same as in the legends for Figs. S2-S4.

Supplementary Figure S6

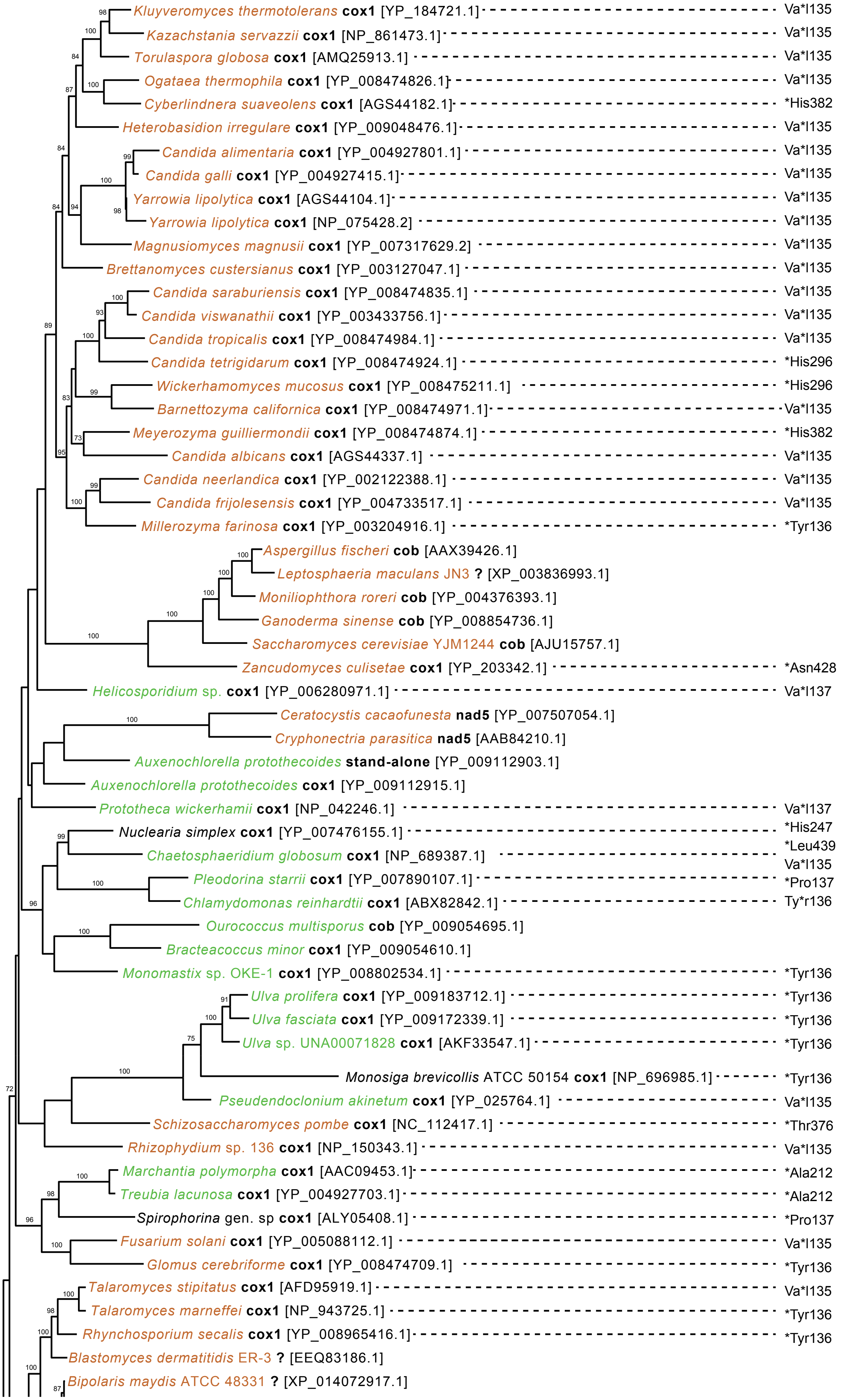

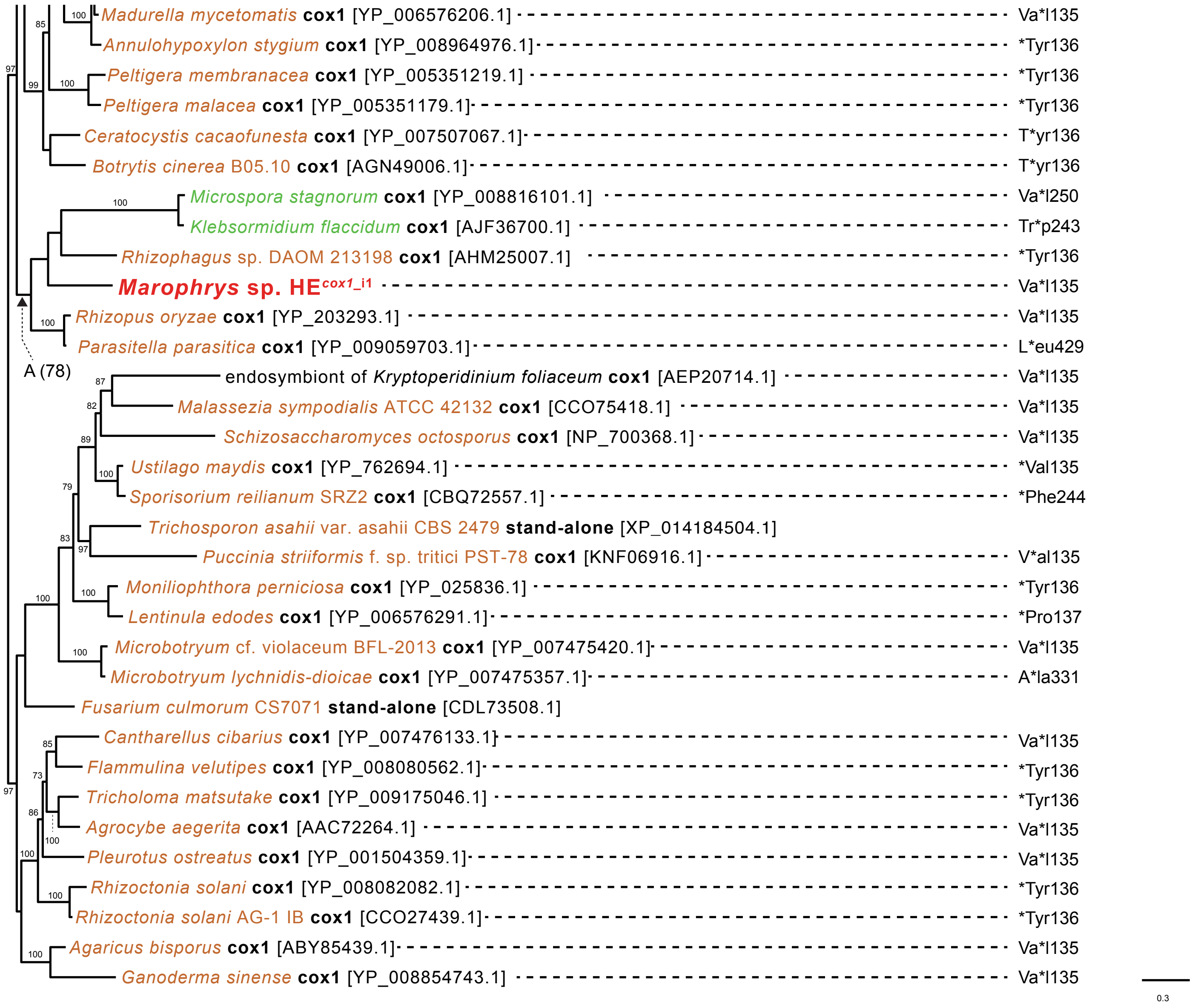

Supplementary Figure 6. Unrooted maximum-likelihood (ML) tree of homing endonuclease (HE) sequences including HE^{cox1_i1}. A phylogenetic analysis was performed on 92 sequences with 271 amino acid positions under the WAG + F + G4 substitution model. Thekey node to infer the origin of the first intron in the *Marophrys cox1*_b locus (*cox1*_i1) and its HE (HE^{cox1_i1}) is labelled with “A” , and the corresponding bootstrap value is shown in parentheses. Other details are the same in the legend for Fig. S2.

Supplementary Figure 7

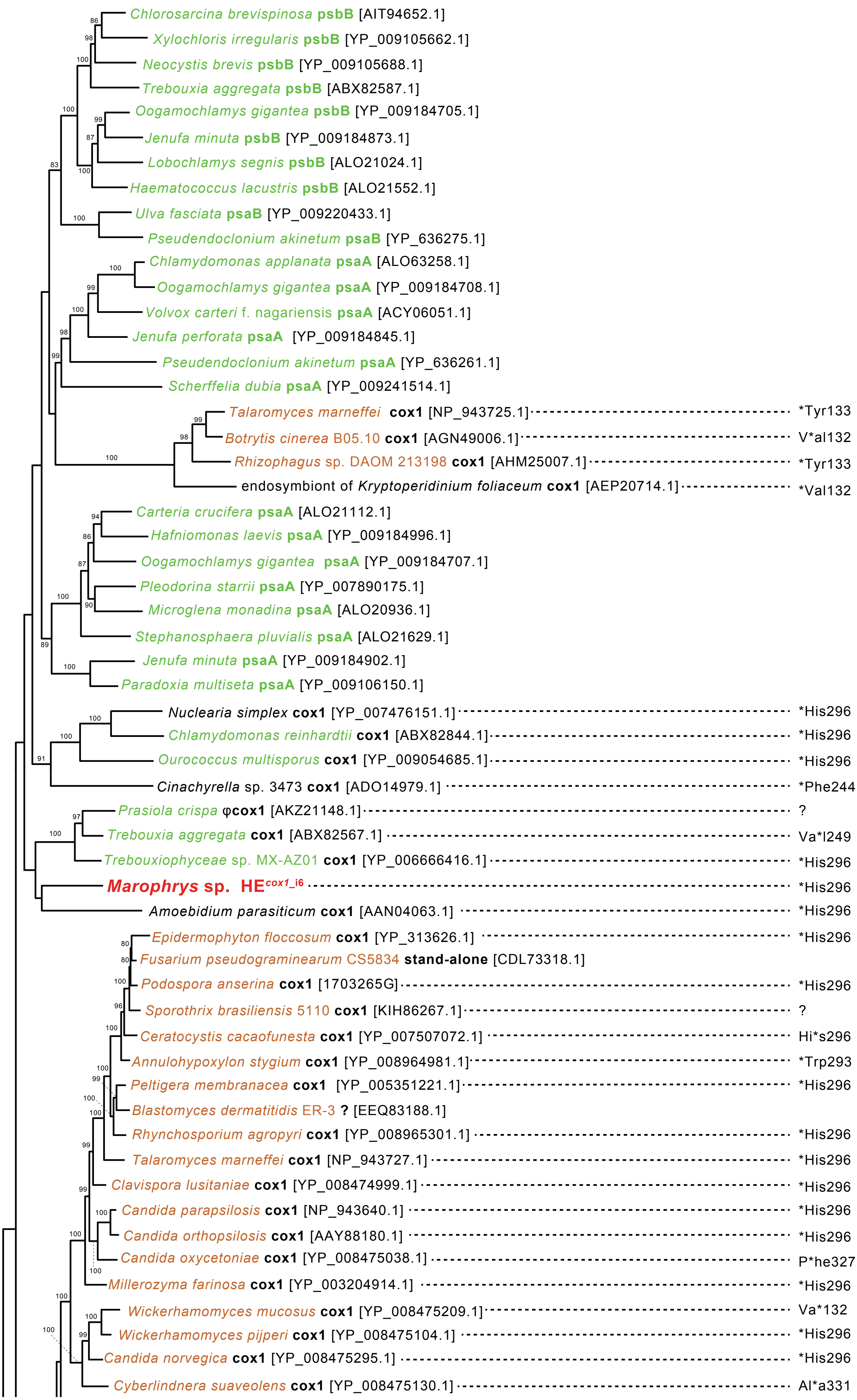

Supplementary Figure 7 (continued)

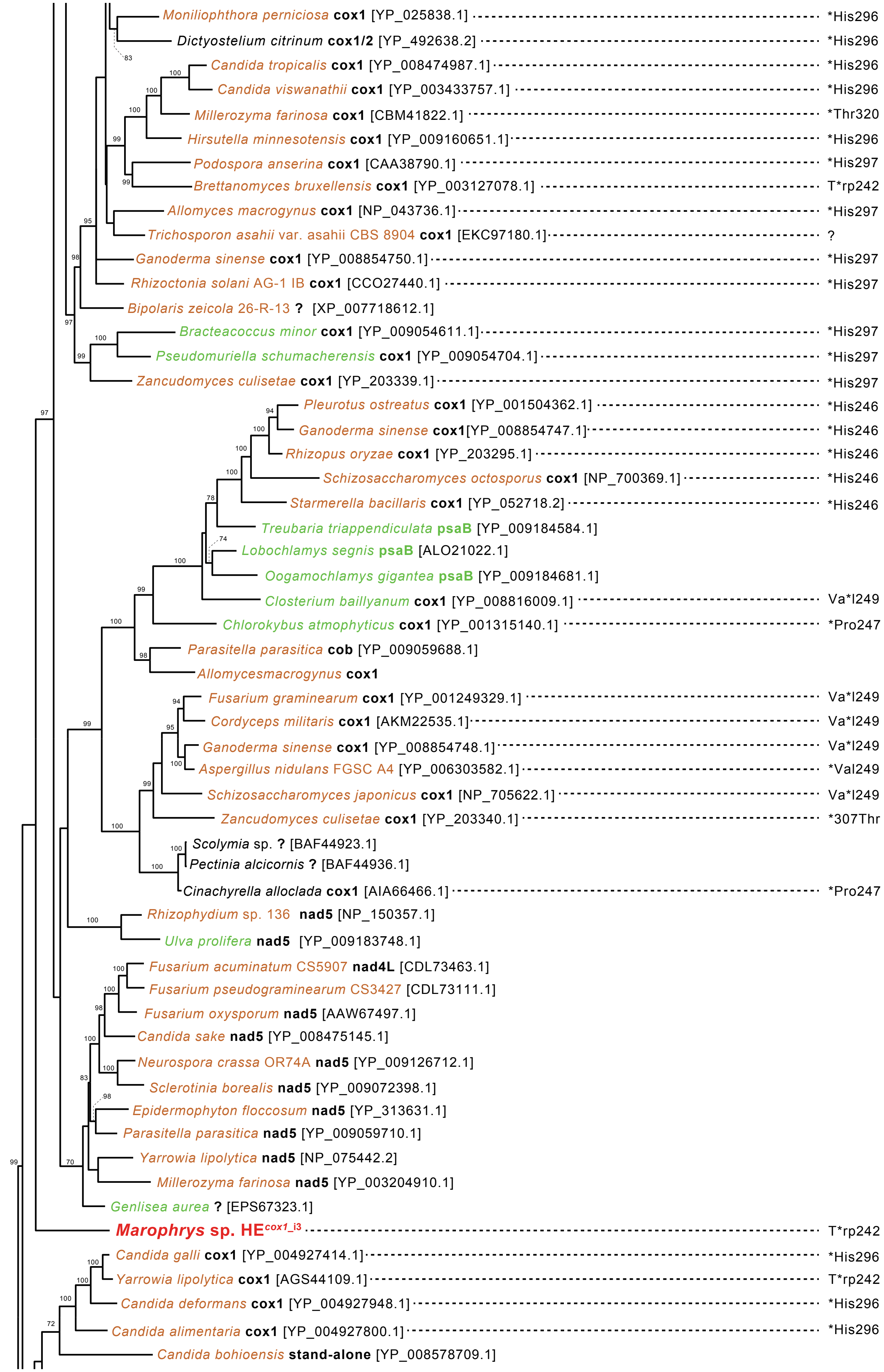

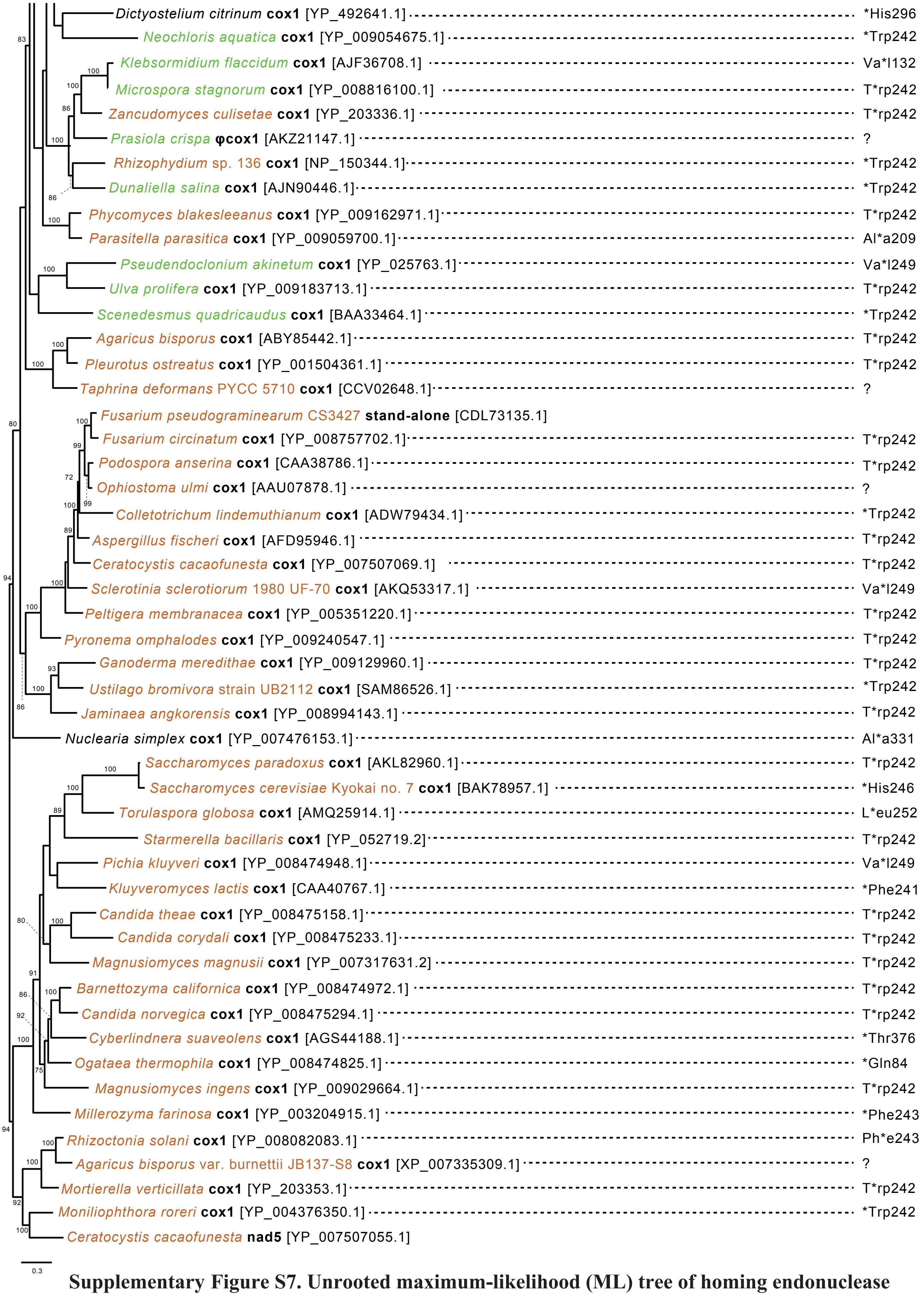

Supplementary Figure S7. Unrooted maximum-likelihood (ML) tree of homing endonuclease (HE) sequences including HE^{cox1_i3} and HE^{cox1_i6}. A phylogenetic analysis was performed on 161 sequences with 213 amino acid positions under the VT + F + I + G4 substitution model. Other details are the same as in the legend for Fig. S2.

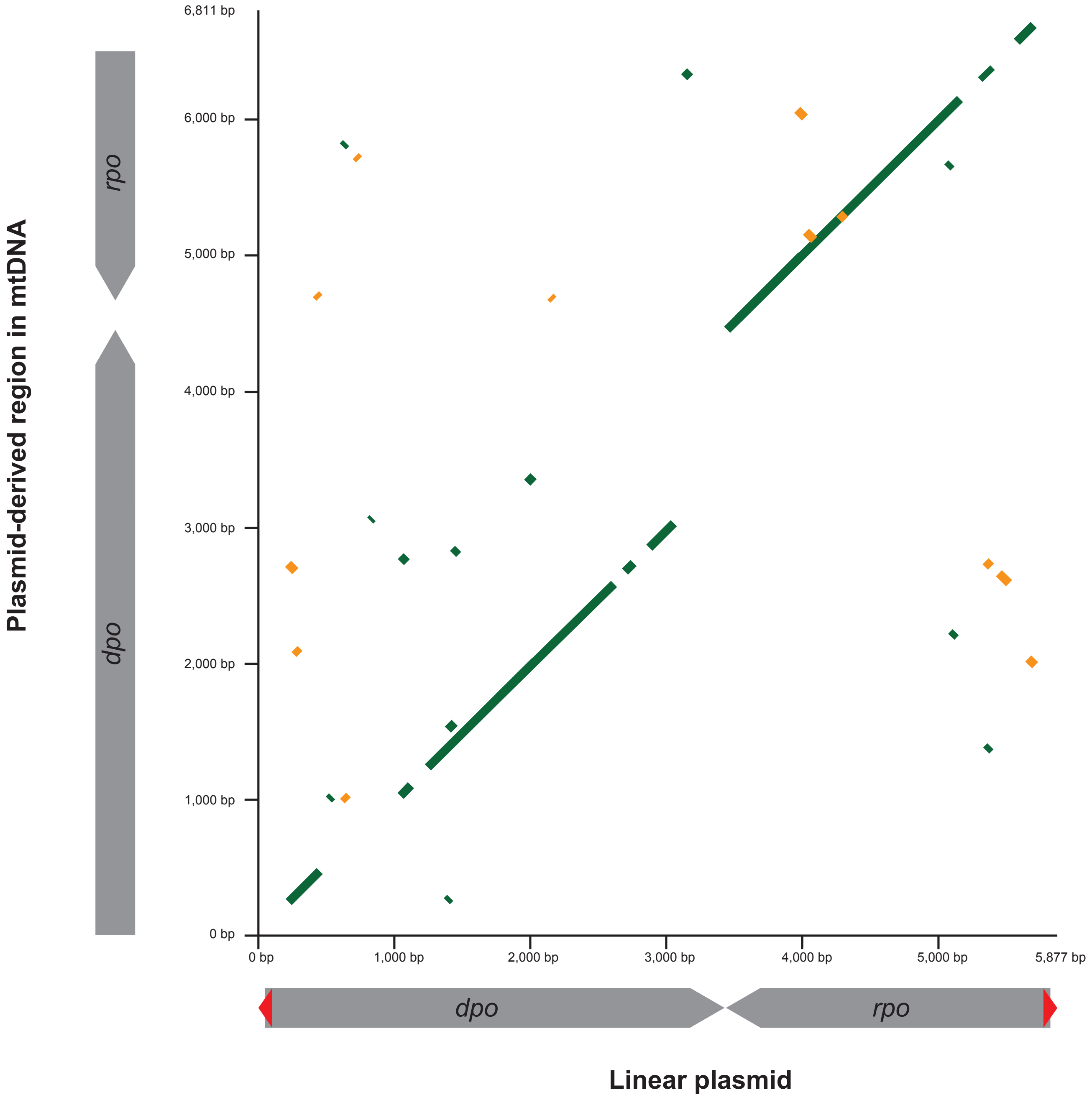

Supplementary Figure 8. Dot plot comparison of the linear plasmid and the plasmid-derived region in the mtDNA. A dot plot was drawn based on the result of YASS[1] analysis with default parameters. The x- and y-axes show the linear plasmid and the plasmid-derived region of the *Marophrys* mtDNA, respectively. Gray arrows along the axes represent genes and their orientation. Inverted repeats in the linear plasmid are depicted as red triangles. Green lines represent forward/forward match in both sequences, whereas the orange ones correspond to the alignment between the reverse complement of the one sequence and the forward of the other.

[1]Noé L. and Kucherov G. 2005, YASS: enchancing the sensitivity of DNA similarity search, Nucleic Acids Res., 33:W540-3

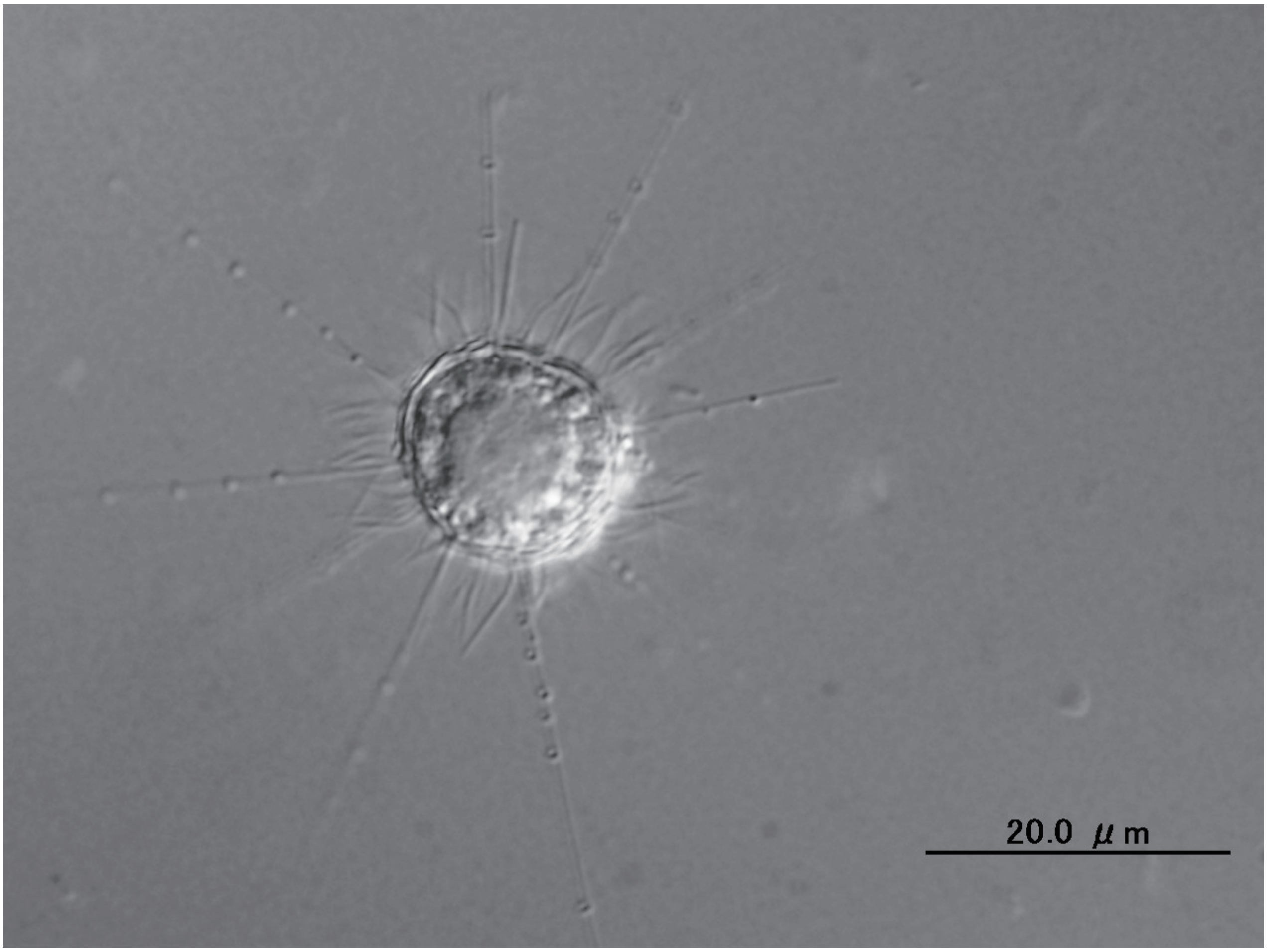

Supplementary Figure 9. Light micrograph of a centrohelid heliozoan *Marophrys* sp. strain SRT127.

Supplementary Table 1: Codon usage in the mitochondrial genome of *Marophrys* sp. strain SRT127

**Codon**	**AA**	**tRNA anticodon**	**Codon**	**AA**	**tRNA anticodon**	**codon**	**AA**	**tRNA anticodon**	**codon**	**AA**	**tRNA anticodon**
UUU	Phe (F)	GAA	UCU	Ser (S)	UGA	UAU	Tyr (Y)	GUA	UGU	Cys (C)	GCA
UUC			UCC			UAC			UGC		
UUA	Leu (L)	—[2]	UCA			UAA	Stop	—[1]	UGA	Trp (W)	—[2]
UUG		CAA	UCG			UAG			UGG		CCA
CUU		UAG	CCU	Pro (P)	UGG	CAU	His (H)	GUG	CGU	Arg (R)	—[2]
CUC			CCC			CAC			CGC		
CUA			CCA			CAA	Gln (Q)	UUG	CGA		
CUG			CCG			CAG			CGG		
AUU	Ile (I)	GAU	ACU	Thr (T)	—[2]	AAU	Asn (N)	GUU	AGU	Ser (S)	GCU
AUC			ACC			AAC			AGC		
AUA			ACA			AAA	Lys (K)	—[2]	AGA	Arg (R)	UCU
AUG	Met (M)	CAU	ACG			AAG			AGG		
GUU	Val (V)	UAC	GCU	Ala (A)	UGC	GAU	Asp (D)	GUC	GGU	Gly (G)	UCC
GUC			GCC			GAC			GGC		
GUA			GCA			GAA	Glu (E)	UUC	GGA		
GUG			GCG			GAG			GGG		

[1] No tRNA for stop codons

[2] tRNA for UUA, UGA, ACN, AAR and CGN (R= A or G and N = A, C, G or T) were not found in the mtDNA

Supplementary Table 2: PCR primers used for confirming intron splicing.

Gene	Name	Direction	Sequences (5′-3′)
atp1	**atp1F**	forward	GAAGCTGCACAGCTAACTGACTTC
	**atp1R**	reverse	CATTGTACAACCTGCGAACGGTG
cob	**cobF**	forward	CAGGTATCGTTGGAACTTGC
	**cobR**	reverse	TACCAGCCAAGTGTAAGCTG
cox1	**cox1F**	forward	GAGCACATTATGCGCGATATCG
	**cox1R**	reverse	GATGCGCCTGCAATAGCAAAG
nad5	**nad5F**	forward	GAGCTGTTGGTAAATCTGCTC
	**nad5R**	reverse	AAGAACCAATGTGCTCGGTGCG
rnl	**rnlF**	forward	GATAGTGAACACAGTACCGTAAG
	**rnsR**	reverse	ATACGTAGCTACTCGACATTGC
rns	**rnsF**	forward	GAGGAATCTTGGACAATGAGCG
	**rnsR**	reverse	CAGCTTCATGCTTTCGAGTTGCAG
